# Spatial transcriptomics reveals molecular differences associated with malignant transformation in oral epithelial dysplasia

**DOI:** 10.3389/fimmu.2026.1817749

**Published:** 2026-07-10

**Authors:** Naren Raja, Harsh B. Pathak, Amrita Mitra, Sufi Mary Thomas, Yong Wang, Tanya Marie Gibson, Rong Wang

**Affiliations:** 1 Department of Oral and Craniofacial Sciences, School of Dentistry, University of Missouri-Kansas City, Kansas City, MO, United States; 2 Pathology and Laboratory Medicine, University of Kansas Medical Center, Kansas City, KS, United States; 3 Otolaryngology-Head and Neck Surgery, University of Kansas Medical Center, Kansas City, KS, United States; 4 Department of Oral Pathology, Radiology and Medicine, School of Dentistry, University of Missouri-Kansas City, Kansas City, MO, United States

**Keywords:** bioinformatics, GeoMx digital spatial profiling, malignant transformation, oral cancer, oral epithelial dysplasia, spatial transcriptomics

## Abstract

**Background:**

Oral epithelial dysplasia (OED) is a precancerous oral lesion with variable risk of progression to oral squamous cell carcinoma (OSCC). The molecular basis underlying this progression remains incompletely understood. To address this gap, this study applied spatial transcriptomics to characterize benign, OED and OSCC biopsies with a focus on the comparison between transforming and non-transforming OED.

**Methods:**

Spatial transcriptomic profiling was performed on 13 benign, 15 OED (8 transforming, 7 non-transforming), and 14 OSCC biopsies using the NanoString GeoMx Digital Spatial Profiler. Regions of interest were segmented into epithelial and immune-enriched compartments with morphological markers. Gene expression was measured using the GeoMx Cancer Transcriptome Atlas (~1, 800 genes) and differentially expressed genes (DEGs) were identified using linear mixed-effects modeling. Exploratory bioinformatic analyses were performed to provide biological context.

**Results:**

Comparison of OED with and without transformation identified a limited set of 11 epithelial DEGs, including genes associated with antigen presentation and interferon signaling (e.g., *B2M*, *STAT1*, and *CD74*), while no significant DEGs were detected in immune-enriched regions. Pathway analyses indicated enrichment of immune- and interferon-related processes. Given the modest sample size and targeted gene panel, these findings should be considered exploratory in nature.

**Conclusions:**

This study provides spatially resolved, exploratory insights into molecular differences between OED lesions with distinct clinical outcomes. The results suggest altered epithelial-immune interactions in transforming lesions, though these findings require validation. Spatial transcriptomics may offer a useful framework for investigating early molecular changes in oral carcinogenesis.

## 1 Introduction

Oral cancer is a major global health burden, with an estimated 377, 713 new cases and 177, 757 deaths reported in 2020, predominantly in low- and middle-income countries (1). More than 90% of oral cancers are classified as oral squamous cell carcinomas (OSCC). Despite advancement in cancer treatment, the overall five-year survival rate for OSCC remains low at 50% to 60% (2–4).

Oral carcinogenesis is a complex, multifactorial, and multistep process that typically begins with benign hyperplasia/hyperkeratosis, progresses through oral epithelial dysplasia (OED) and carcinoma *in situ*, and ultimately develops into invasive OSCC (5). OED is a histopathologically diagnosed precancerous lesion that carries an increased risk of malignant transformation (MT) into OSCC. The malignant transformation rate of OED ranges from 5% to 36%, depending on the severity of dysplasia (6). Clinically, OED typically presents as leukoplakia, erythroplakia, or leukoerythroplakia (7). At the molecular level, it remains unclear why some OED lesions progress to OSCC while others don’t, and the mechanisms driving OED progression are still poorly understood (8).

Spatial transcriptomics enables the measurement of messenger RNA (mRNA) expression across different regions of a tissue, allowing analysis of gene activity within its native histological context (9, 10). Advances in imaging, biomarker detection, high-throughput sequencing, and bioinformatics have further enhanced the ability to study complex disease mechanisms, including cancer.

In this study, we employed the NanoString’s GeoMx Digital Spatial Profiling (DSP) platform to investigate formalin-fixed paraffin-embedded human oral tissue biopsies from benign, OED and OSCC cases. Importantly, OED samples included patients with clinical follow-up, enabling comparison between non-transforming (OED-NT) and transforming (OED-T) lesions. We performed spatial transcriptomic analysis and downstream bioinformatic analyses across multiple group comparisons, with a primary focus on gene expression differences between OED-NT and OED-T.

To the best of our knowledge, this is among the first studies to apply spatial transcriptomic techniques to directly compare these clinically distinct OED groups in human tissues. Our findings provide exploratory insights into molecular differences associated with oral carcinogenesis and may inform future studies aimed at improving risk stratification of potentially malignant oral lesions and early detection of oral cancer.

## 2 Materials and methods

**Figure 1:**
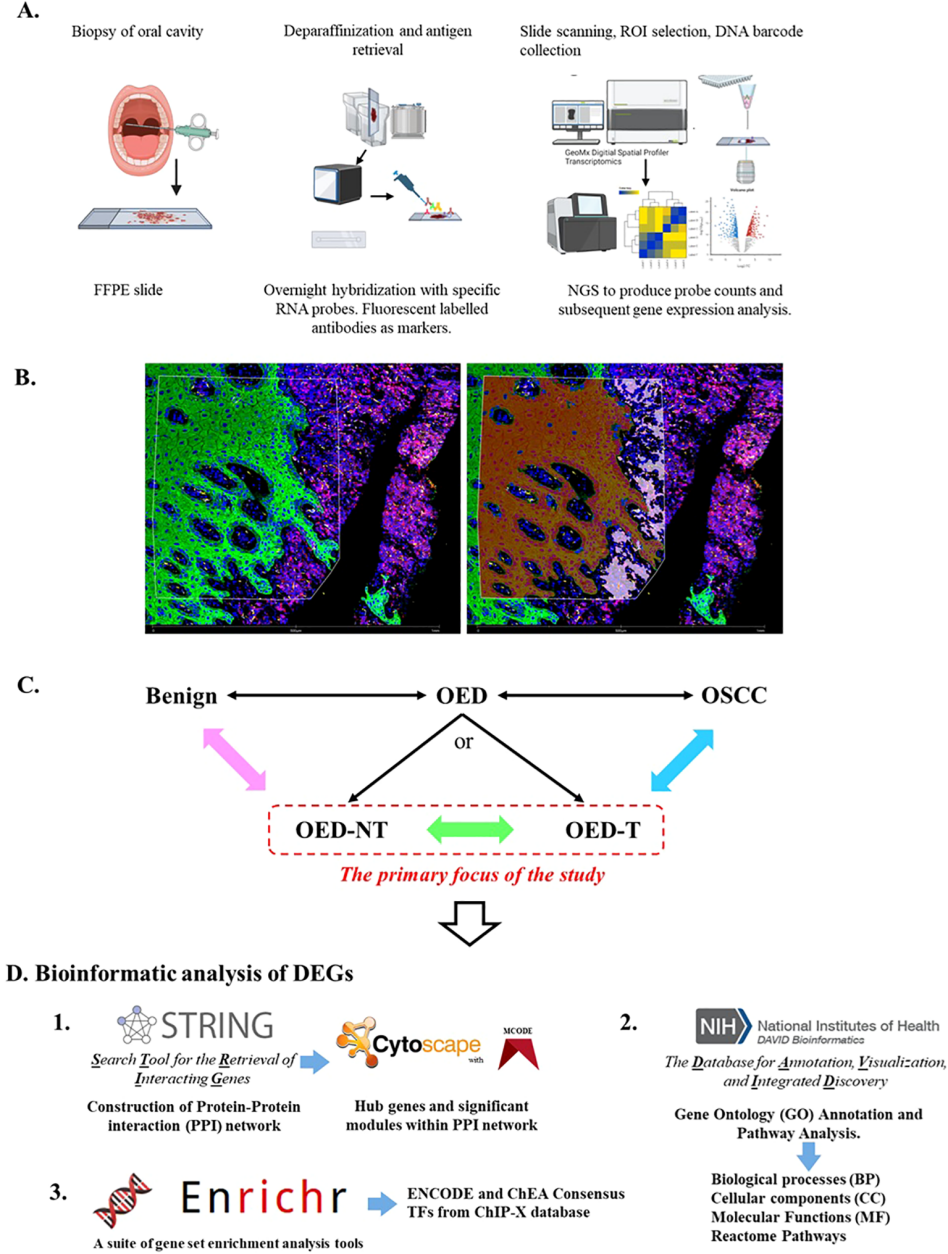
Overall workflow of the study: **(A)** Schematic illustration of the GeoMx DSP experiment (created with BioRender). **(B)** Representative OED tissue sections stained with morphological markers panCK (green), CD45 (red), CD163 (yellow), and Syto-13 (blue). Regions of interest (ROIs) were selected and segmented into panCK^+^ epithelial and CD45^+^ immune-enriched regions for spatial profiling. **(C)** Schematic illustration of comparison groups, with a focus on OED-NT vs. OED-T comparison. **(D)** Differentially expressed genes were subjected to exploratory downstream analyses, including protein–protein interaction (PPI) network analysis (STRING and Cytoscape with MCODE), gene ontology (GO) and pathway enrichment (DAVID), and transcription factor enrichment analysis (Enrichr).

**Figure 1** illustrates the overall workflow of the study.

### 2.1 Sample preparation

This study was approved by the Institutional Review Board of University of Missouri at Kansas City (UMKC) for the use of archived human oral tissues. The pathology database of the UMKC School of Dentistry was searched to identify suitable biopsy samples for the investigation. The identification of benign and OSCC cases was based on histopathological diagnoses. OED cases were classified based on clinical follow-up. Transforming OED (OED-T) cases were defined as lesions initially diagnosed as dysplasia with subsequent OSCC diagnosis in the same patient. Non-transforming OED (OED-NT) cases were defined as dysplastic lesions that remained clinically non-progressive within a minimum follow-up period of two years, based on the reported average OED transformation time of 1.82 ± 1.55 years (11). We acknowledge that the timeline for malignant transformation varies significantly, and that classification of OED-NT reflects clinical behavior within the observation window rather than definitive non-transformation, as some lesions may represent slow-progressing cases. However, given the limited availability of OED cases with adequate follow-ups, this approach enables a practical cohort size while allowing investigation of early molecular differences associated with divergent clinical outcomes. A total of 42 archived oral biopsy samples were included, including 13 benign, 15 OED, and 14 OSCC biopsies. Among the OED biopsies, 8 were classified as OED-T and 7 as OED-NT). Detailed demographic and clinical characteristics of the sample cohort, including sex, birth year, biopsy site, histopathological diagnosis, and clinical presentation, are provided in **Supplementary Material 1.**

The biopsy blocks were cut into 5-µm sections using a manual microtome (Leica RM2125, RTS, Leica Biosystems Inc. Buffalo Grove, IL, USA). Two adjacent sections were placed on positively charged glass slides for Hematoxylin and Eosin staining and DSP (**Figure 1A**).

### 2.2 GeoMx digital spatial profiling

Spatial transcriptomic profiling was performed using the GeoMx DSP platform (Bruker NanoString Technologies) with the human Cancer Transcriptome Atlas (CTA) panel to measure the expression of ~1, 800 genes related to tumor biology. This technology has been widely validated across various cancer studies (12–14). While this targeted panel enables robust profiling of key oncogenic pathways, it does not capture the full transcriptome; therefore, downstream interpretations are limited to the genes included in this panel. Standard GeoMx protocols were used for slide preparation, *in situ* probe hybridization, immunofluorescent staining, oligonucleotide collection, and data processing. Specifically, slides were stained with fluorescently conjugated antibodies targeting panCK (epithelial marker), CD45 (immune marker), CD163 (macrophage marker), and Syto-13 (nuclear stain).

Regions of interest (ROIs) were selected by a board-certified oral pathologist based on review of corresponding H&E-stained sections. Selection criteria aimed to capture morphologically representative areas of epithelial (benign, dysplasia, and OSCC) and adjacent stromal regions, while avoiding artifacts such as necrosis, tissue damage, or poor morphological preservation. A minimum of three ROIs per sample were selected in a standardized manner to account for intra-lesional heterogeneity, with a fourth ROI included where tissue availability and morphological diversity permitted. ROIs were selected to represent distinct regions within each lesion where possible, while maintaining consistency across samples. Within each ROI, segmentation was performed to designate panCK^+^ epithelial regions, CD45^+^ immune-enriched regions, and panCK^-^/CD45^-^/CD163^-^ stromal/non-immune regions (**Figure 1B**). We note that these compartments represent broad tissue domains and likely contain heterogeneous cell populations; therefore, interpretations were made at the compartment level rather than at specific immune cell subtype resolution. Photo-cleavable oligonucleotides from each segmented region were collected into individual wells of a 96-well plate and gene expression was quantified using next-generation sequencing (NGS) on an Illumina NextSeq 550 platform.

### 2.3 Data analysis

Three main comparison groups were analyzed, including OED-NT vs. Benign, OED-T vs. OED-NT, and OSCC vs. OED-T. Additional comparisons, including OED (combined OED-T and OED-NT) vs. Benign, OSCC vs. OED, and OSCC vs. Benign, were also conducted for completeness and reference. While analyses were performed across the full dataset, the primary focus of this study was the OED-T vs. OED-NT comparison, as it directly relates to investigating early molecular differences associated with divergent malignant transformation outcomes within dysplastic lesions (**Figure 1C**). These analyses were cross-sectional and do not represent a longitudinal or trajectory of disease progression. Results from additional analyses are provided in **Supplementary Materials 2–15**.

#### 2.3.1 Differentially expressed gene analysis

Following sample collection and sequencing, FASTQ files were processed using the GeoMx NGS Pipeline (v2.3.3.10) to generate digital count conversion files, which were subsequently analyzed in the GeoMx DSP Analysis Suite (v3.0). Quality control (QC) filtering was performed at the segment level using GeoMx criteria, including sequencing depth, alignment rate, sequencing saturation, surface area, and nuclei count. Gene filtering was based on the limit of detection (LOD), retaining genes expressed in ≥ 5% of segments. Data were normalized using third-quartile (Q3) normalization to account for technical variability across segments. Differential expression analysis was performed using a linear mixed-effects model framework implemented within the GeoMx DSP Analysis Suite. For each gene, group status (e.g., OED-T vs. OED-NT) was included as a fixed effect, and patient ID was included as a random intercept to account for multiple ROIs per case. To account for multiple hypothesis testing across ~1, 800 genes, Benjamini-Hochberg (BH) correction was applied. Statistical significance was defined as an adjusted p-value (false discovery rate, FDR) < 0.05, in combination with an absolute fold change ≥ 1.5 (log_2_ fold change ≥ ± 0.58). Given the modest sample size and subtle transcriptional differences between OED-NT and OED-T, this stringent threshold yielded a limited number of significant genes. To further explore potential biological signals, additional analyses using nominal p-values (p < 0.05) were conducted and are presented as exploratory and hypothesis-generating. Adjusted p-values are reported alongside nominal p-values for transparency. Volcano plots were generated by plotting the –log_10_(p) against the log_2_ fold change for each gene to visualize the DEGs. The resulting DEG lists were subsequently used for downstream exploratory bioinformatic analyses.

#### 2.3.2 Construction of protein-protein interactions network construction

Using the list of DEGs, protein-protein interactions, including both physical and functional interactions, were identified via the STRING (Search Tool for the Retrieval of Interacting Genes) database (http://string-db.org/) (15). The PPI network was constructed by integrating both upregulated and downregulated genes, applying a minimum required interaction score of 0.4 to define significant protein interactions. The resulting PPI network map was then imported into Cytoscape (version 3.10.3; https://cytoscape.org/) (16) for further visualization and analysis (**Figure 1D-1**).

#### 2.3.3 PPI network analysis using MCODE

In Cytoscape, the MCODE addon was utilized to identify densely connected or highly functional modules within the PPI network. Key clusters were detected using the following parameters: degree cutoff of 2, node score cutoff of 0.2, k-core of 2, and a maximum depth of 100. Self-interactions or loops were excluded from the analysis. For each identified cluster, the number of nodes, edges and the MCODE score were recorded.

#### 2.3.4 Gene ontology and pathway enrichment analysis

**Figure 2:**
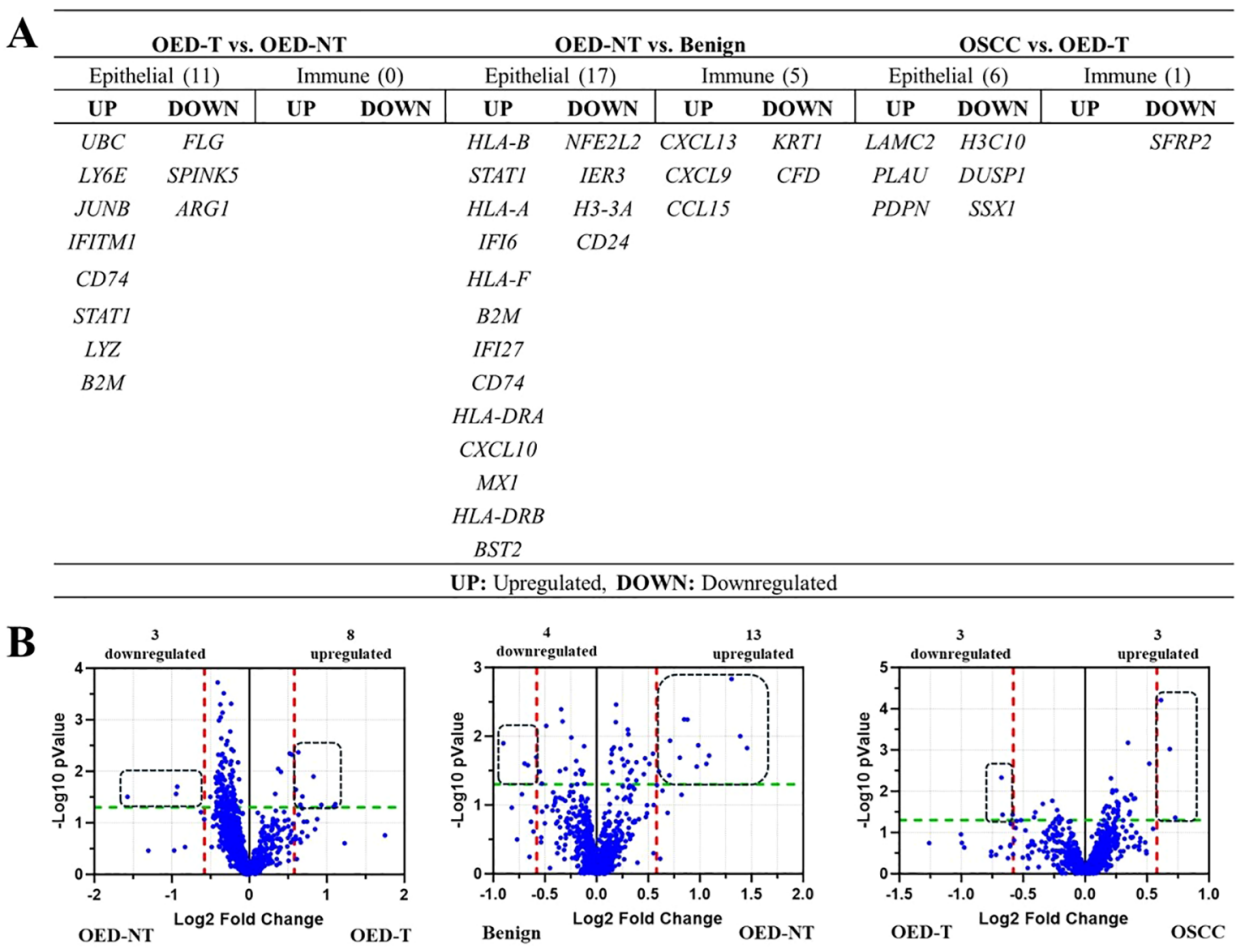
Differential gene expression analysis across the three main comparison groups. **(A)** Summary of epithelial and immune-enriched DEGs across the three main comparison groups. **(B)** Volcano plots of epithelial gene expression, showing log_2_ fold change vs. –log_10_(p), with DEGs highlighted in dashed boxes.

The functional roles of DEGs were analyzed using the DAVID (Database for Annotation, Visualization and Integrated Discovery) tool (https://davidbioinformatics.nih.gov/home.jsp) (17), focusing on gene ontology (GO) categories (biological processes, molecular functions, and cellular components) and signaling pathways (**Figure 1D-2**). Given the limited number of DEGs identified in OED-NT and OED-T related comparisons, enrichment analyses were performed using a nominal significance threshold of p < 0.05 and are presented as exploratory. For broader baseline comparisons among benign, OED, and OSCC, BH correction was applied, with an adjusted p-value (FDR) < 0.05. This approach ensured that our reference group was anchored by the most reliable biological signals. Both Reactome and KEGG (Kyoto Encyclopedia of Genes and Genomes) pathways were evaluated for these analyses.

#### 2.3.5 Transcription factor enrichment analysis

**Figure 3:**
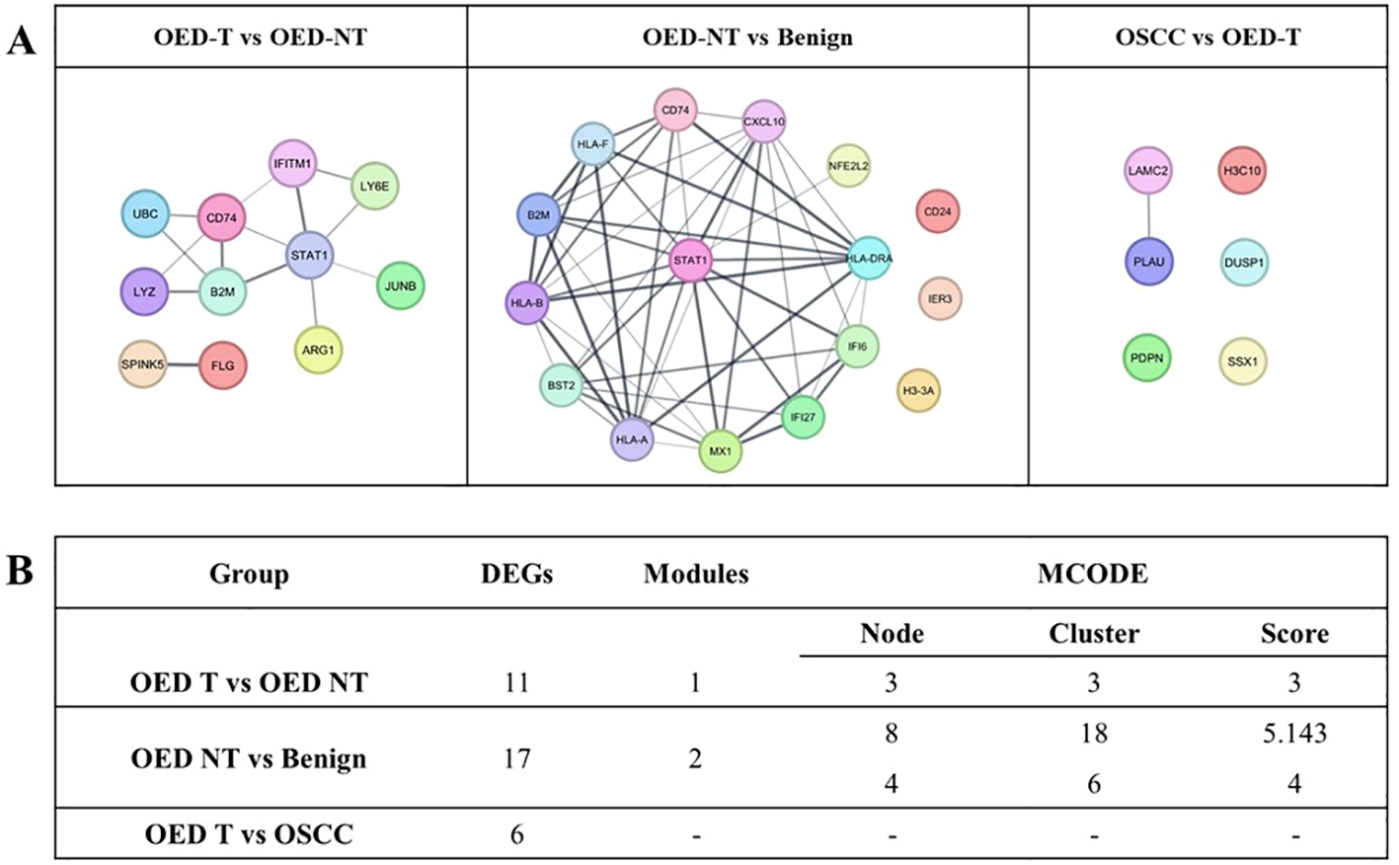
**(A)** PPI networks generated by the STRING database for the three main comparison groups. **(B)** Summary of number of nodes, edges and the MCODE score for these comparisons.

Potential TFs regulating the DEGs were identified using the online tool Enrichr (https://maayanlab.cloud/Enrichr) (18), using the ENCODE (https://www.encodeproject.org/) and ChEA consensus datasets from the ChIP-X database. An adjusted p <0.05 was used as the cutoff (**Figure 1D-3**). The resulting list of TFs represents potential factors contributing to the up- or down-regulation of DEGs in each comparison.

## 3 Results

### 3.1 Identification of DEGs

DEGs identified across the three main comparisons are summarized in **Figure 2A**. The OED-NT vs. Benign comparison identified 17 epithelial and 5 immune DEGs; the OED-T vs. OED-NT comparison identified 11 epithelial and no significant immune DEGs; the OSCC vs. OED-T comparison identified 6 epithelial and one immune DEG. **Figure 2B** shows the volcano plots for DEG analyses for the three comparisons. In the OED-T vs. OED-NT comparison, 11 identified DEGs included 8 upregulated and 3 downregulated genes. Given the limited number of DEGs identified in immune-enriched regions, downstream bioinformatic analyses focused primarily on epithelial DEGs.

### 3.2 PPI network construction and analysis

PPI networks were constructed for each DEG set using the STRING database. As shown in **Figure 3A**, the OED-T vs. OED-NT comparison generated a relatively small network consisting of 11 nodes and 14 edges, with *B2M* identified as the central node in both STRING and MCODE analyses (**Figure 3B**). Among the downregulated genes, *ARG1* showed co-expression with *STAT1*, whereas *SPINK5* and *FLG* expressed independently.

The OED-NT vs. Benign comparison produced a more complex PPI network comprising 16 nodes and 49 edges. While all upregulated genes were involved in the main network, only one downregulated gene *NFE2L2* showed co-expression with *STAT1*. MCODE analysis identified two network modules with *HLA-F* and *CXCL10* as seed node. In contrast, the OED-T vs. OSCC comparison showed a less connected network structure, with several genes exhibiting limited or no interactions.

### 3.3 GO annotation and pathway analysis

**Figure 4:**
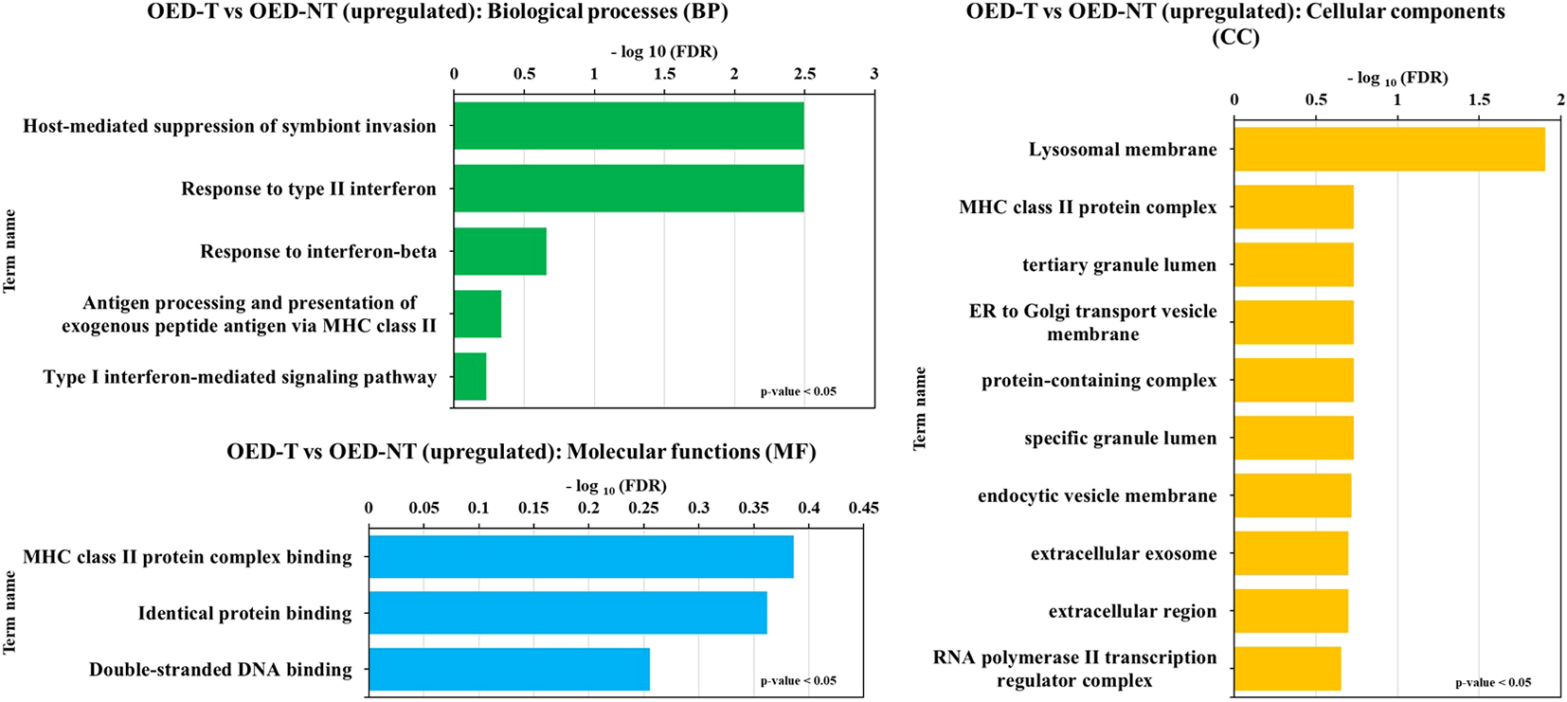
Gene ontology (GO) enrichment analysis of epithelial DEGs in the OED-T vs. OED-NT comparison. Biological processes, cellular components and molecular functions identified from GO analysis of epithelial DEGs in the OED-T vs. OED-NT comparison.

GO analyses on epithelial DEGs in the OED-T vs. OED-NT comparison identified five significant biological processes, ten cellular components, and three molecular functions (p < 0.05) (**Figure 4**). Enriched cellular components were primarily associated with lysosomal membrane and other membrane-related structures. Identified biological processes included interferon-related responses and host-mediated suppression of invasion, while molecular functions included MHC class II protein complex binding and identical protein binding.

**Figure 5:**
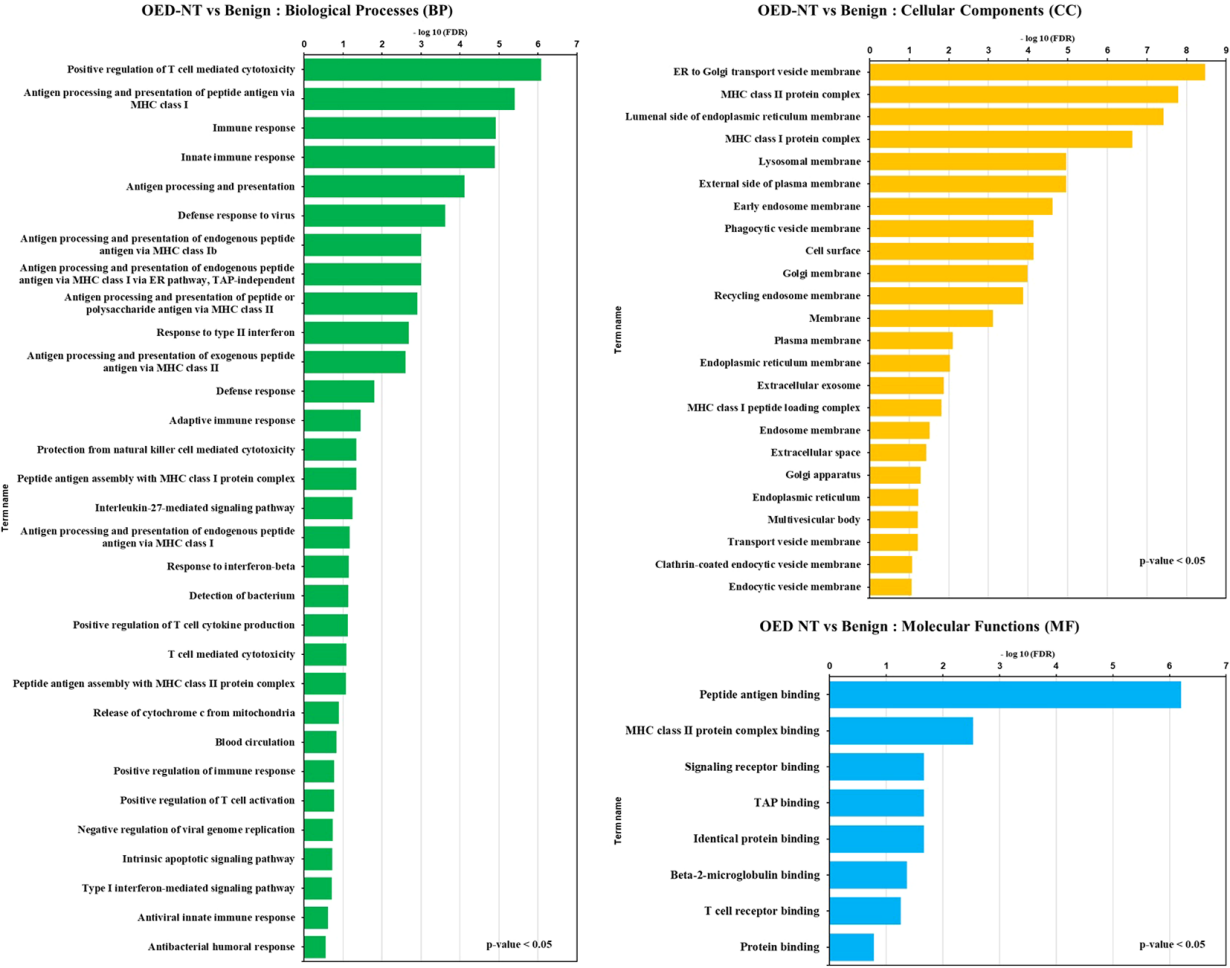
Gene ontology (GO) enrichment analysis of epithelial DEGs in the OED-NT vs. Benign comparison. Enriched biological processes, Cellular components and Molecular functions identified through enrichment analysis of DEGs between OED-NT and Benign groups.

The OED-NT vs. Benign comparison identified 31 biological processes, 24 cellular components, and 8 molecular functions (**Figure 5**). Enriched biological processes were predominantly related to innate and adaptive immune responses, antigen processing and presentation, especially via MHC class I and II pathways. Cellular components comprised of membrane-associated organelles and peptide-loading complexes, whereas molecular functions were mainly associated with antigen and protein complex binding. In contrast, the OED-T vs. OSCC comparison identified only one significant biological process, positive regulation of cell migration.

**Figure 6:**
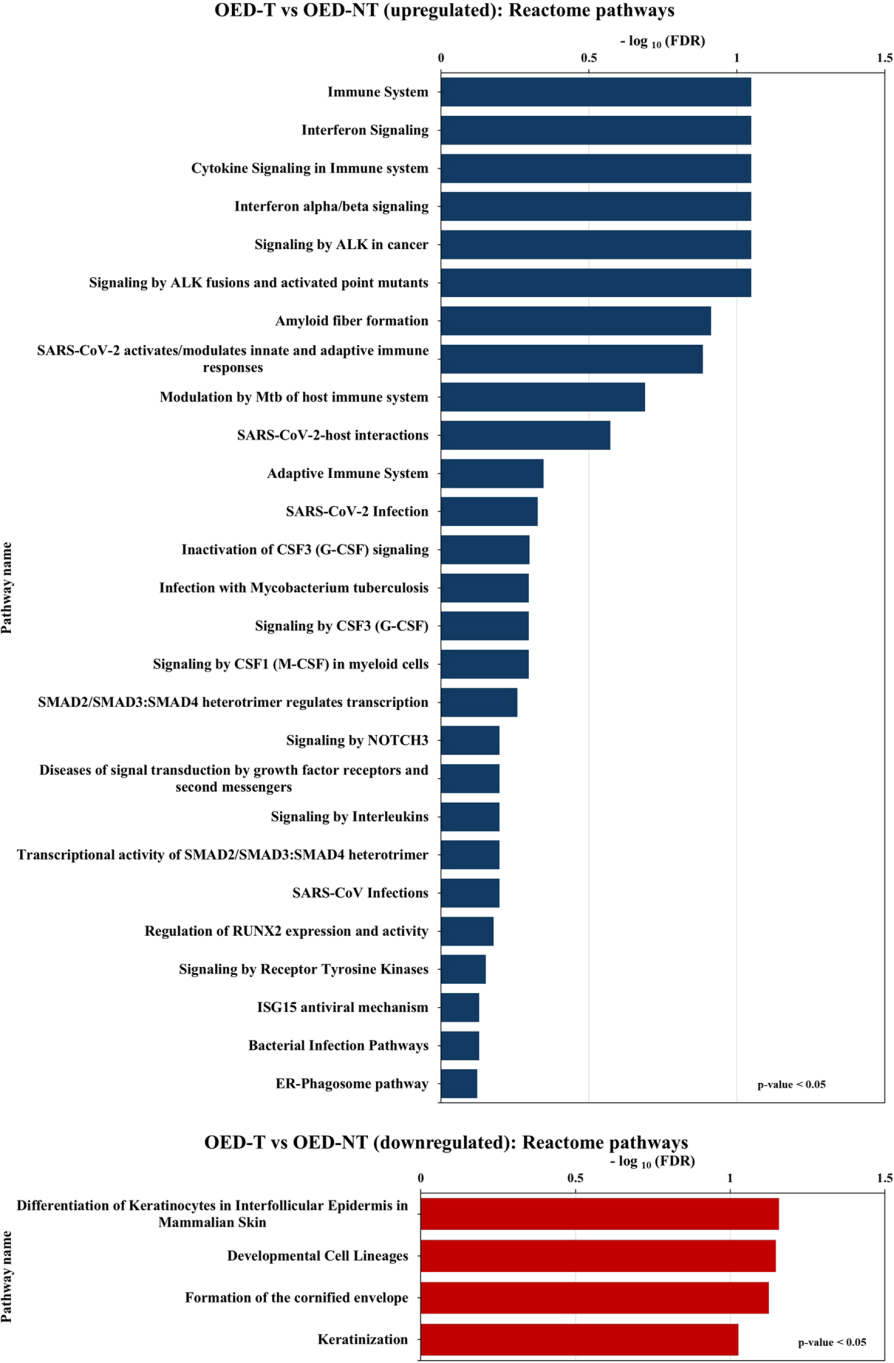
Reactome pathways enriched by the upregulated and downregulated genes in the OED-T vs. OED-NT comparison.

In the DAVID analysis, the Reactome database was selected over KEGG due to its broader coverage of human biological pathways, more diverse biochemical reactions and molecular events. In the OED-T vs. OED-NT comparison (**Figure 6**), enriched pathways were primarily related to immune system processes, cytokine and interferon signalling pathways, and host responses to bacterial and viral infection. In contrast, pathways associated with keratinocyte differentiation and cornified envelope formation were downregulated in OED-T lesions.

**Figure 7:**
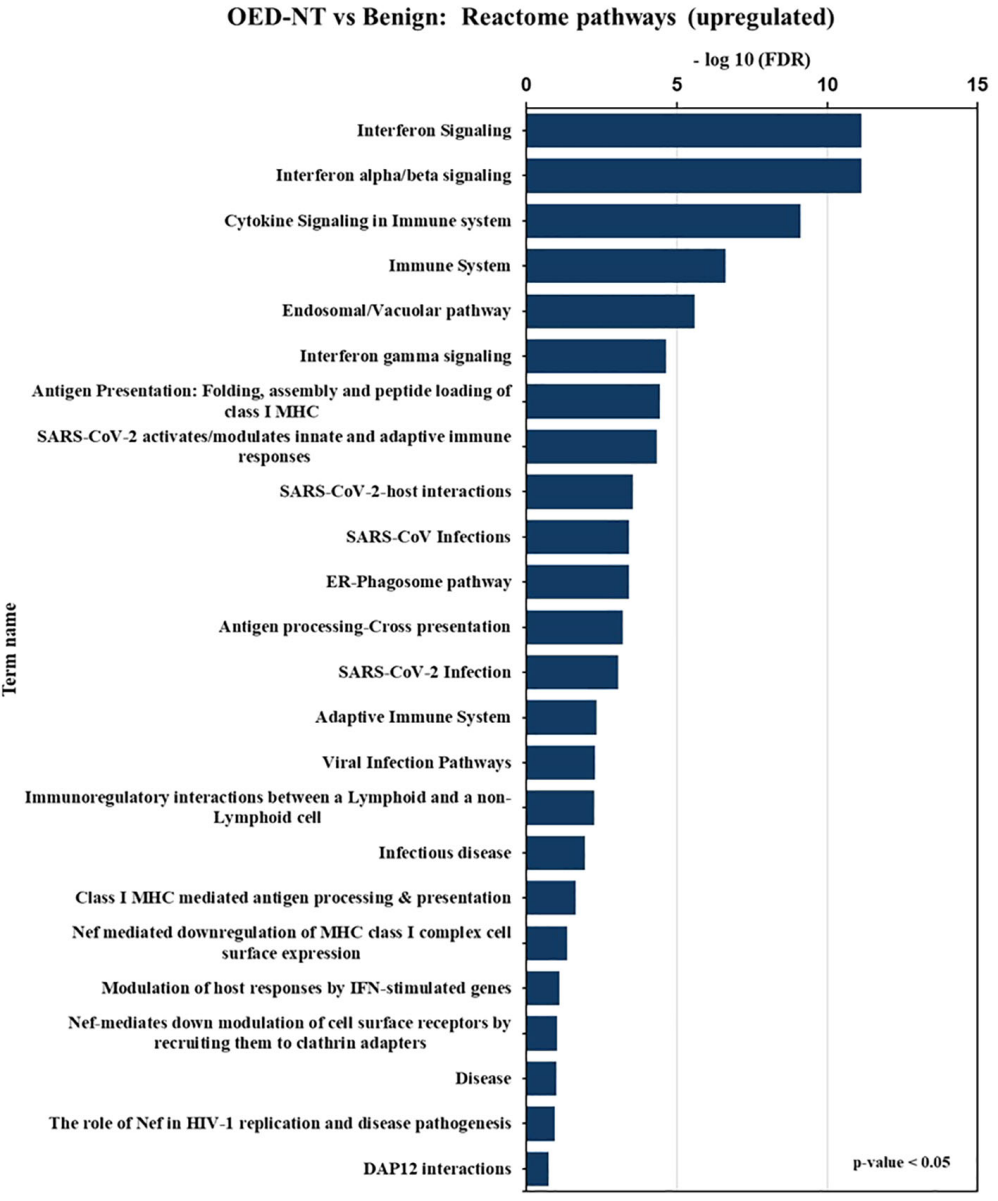
Reactome pathways enriched by the upregulated genes in the OED-NT vs. Benign comparison.

The OED-NT vs. Benign comparison identified 24 enriched pathways among upregulated genes, whereas no significant pathways were associated with downregulated genes (**Figure 7**). These pathways were predominantly related to immune activation, signaling cascades, and host responses to infection. Due to the limited number of DEGs identified in the OSCC vs. OED-T comparison, no significant hits were obtained in the subsequent gene enrichment analysis (p < 0.05).

### 3.4 Transcriptional factors identified from DEGs

TF analysis was conducted separately for upregulated and downregulated DEGs to distinguish potential activating and repressive regulators. **Table 1** summarizes eight significant TFs of upregulated DEGs in the OED-T vs. OED-NT comparison. Among these, RELA and RFX5 each regulates four target genes: RELA targets *CD74*, *STAT1*, *JUNB*, and *B2M*, while RFX5 targets *CD74*, *UBC*, *JUNB*, and *B2M*. Additional TFs, including NELFE, STAT3, SPI1, ETS1, IRF1, and BCL3, were associated with two to three target genes. *JUNB* and *B2M* were the most targeted genes, regulated by multiple TFs. No significant TFs were identified for downregulated genes.

**Table 1:** Transcription factors (TFs) identified as upstream regulators of upregulated and downregulated genes in the OED-T vs. OED-NT and OED-NT vss benign comparisons (adjusted p < 0.05).

TFs (source database)	Adjusted p-value	No. of targets	Targets
Upregulated DEGs OED-T vs. OED-NT			
RELA (ENCODE)	0.0010	4	*CD74; STAT1; JUNB; B2M*
RFX5 (ENCODE)	0.0010	4	*CD74; UBC; JUNB; B2M*
NELFE (ENCODE)	0.0015	3	*UBC; JUNB; B2M*
ETS1 (ENCODE)	0.0229	2	*UBC; JUNB*
STAT3 (ENCODE)	0.0259	3	*STAT1; UBC; JUNB*
IRF1 (ENCODE)	0.0393	2	*STAT1; B2M*
BCL3 (ENCODE)	0.0393	2	*STAT1; UBC*
SPI1 (CHEA)	0.0470	3	*UBC; JUNB; B2M*
Upregulated DEGs OED-NT vs. Benign			
IRF1 (ENCODE)	0.0005	4	*STAT1; MX1; IFI6; B2M*
IRF8 (CHEA)	0.0008	4	*CD74; HLA-DRA; HLA-A*
RELA (ENCODE)	0.0019	4	*BST2; CD74; STAT1; B2M*
RFX5 (ENCODE)	0.0365	3	*CD74; IFI27; B2M*
Downregulated DEGs OED-NT vs. Benign			
ETS1 (ENCODE)	0.0136	2	*NFE2L2; IER3*

Given the relatively small DEG sets and the nature of enrichment-based inference, all bioinformatic analyses should be interpreted as exploratory and descriptive. These results are therefore considered hypothesis-generating rather than definitive.

The OED-NT vs. Benign comparison identified four TFs for upregulated DEGs, including IRF1, IRF8, and RELA, each targeting four genes. A single TF was identified for downregulated genes that suppresses *NFE2L2*. In contrast, no significant TFs were identified in the OSCC vs. OED-T comparison, likely due to the limited number of DEGs.

## 4 Discussion

OSCC remains a major head and neck malignancy with limited therapeutic options, highlighting the need for improved early detection and prevention. OED is a recognized precursor to OSCC, but its clinical course is heterogeneous, and histopathological grading alone has limited predictive value for malignant transformation (11, 19, 20). Identifying molecular differences between lesions with divergent clinical outcomes may improve risk stratification and inform prevention strategies (21).

In this study, we applied spatial transcriptomics to compare OED lesions that progressed to invasive OSCC (OED-T) with those that remained clinically stable for at least two years following diagnosis (OED-NT). We acknowledge that this classification reflects observed clinical behavior rather than definitive non-transformation. The analysis is cross-sectional and therefore identifies group-level molecular differences, not a temporal progression trajectory.

Although multiple group comparisons were made, the primary focus was the OED-NT vs. OED-T comparison, which yielded a limited set of 11 epithelial DEGs. Application of multiple testing correction (Benjamini-Hochberg FDR) resulted in very few statistically significant genes, reflecting the modest sample size and subtle transcriptional differences between these groups. Therefore, exploratory analyses based on nominal p-values were used to identify potential signals.

Among the identified genes, *B2M*, *STAT1*, and *CD74* are well-established components of antigen presentation and interferon signaling. Their identification likely reflects both biological relevance and their high connectivity in curated databases. Accordingly, these genes are best interpreted as immune-associated markers of interest, rather than definitive drivers of malignant transformation.

Specifically, *B2M* (Beta-2-microglobulin) encodes the non-polymorphic light chain of the MHC class I (MHC-I), essential for antigen presentation to cytotoxic T lymphocytes. Altered *B2M* expression destabilizes MHC-I complexes, impairs immune surveillance, and promotes tumor immune evasion (22). In OSCC, *B2M* expression has been shown to correlate with larger tumors, node positivity, advanced stage, metastasis, and worse survival (23–25). In this study, we observed increased *B2M* expression in OED-T compared to OED-NT, suggesting that *B2M* upregulation may be associated with early immune escape and malignant transformation in OED.

*STAT1* (Signal Transducer and Activator of Transcription 1) is a key interferon-signaling mediator and plays dual roles in cancer immunity. Upon activation, *STAT1* is translocated to the nucleus to regulate antiviral defense, apoptosis, proliferation and immune surveillance as a tumor suppressor (26, 27). However, chronic activation or dysregulated *STAT1* activity in the tumor microenvironment can paradoxically promote immune evasion by inducing PD-L1 expression, fostering T cell exhaustion, and altering cytokine profiles (28). *STAT1* expression was shown to be enriched in moderate-severe OED and further in early stage OSCC (29). Upregulation of *STAT1* was associated with a proinflammatory macrophage phenotype driven by premalignant changes in oral epithelium (30). *STAT1* expression was elevated along with *STAT3*, *STAT5a* in oral lichen planus tissues compared to normal tissues, consistent with our GO analysis (31). We likewise observed higher *STAT1* in OED-T than OED-NT, suggesting altered epithelial-immune interactions associated with transforming OED.

*CD74* (invariant chain) is required for MHC class II (MHC-II) assembly and antigen presentation to CD4^+^ T cells. *CD74* also serves as a receptor for macrophage migration inhibitory factor (MIF), influencing macrophage polarization toward an immunosuppressive phenotype. *CD74* was also reported to support accumulation and function of tumor-infiltrating regulatory T cells and its level increased when benign oral lesions progressed to OSCC (32, 33). In our study, *CD74* expression was higher in OED-T than in OED-NT, suggesting its potential role associated with OED malignant transformation.

Overall, the upregulation of *B2M*, *STAT1*, and *CD74* in OED-T suggests increased immune- or interferon-associated transcriptional activity within epithelial regions. However, these signals are not specific to malignant transformation and may also reflect inflammatory or broader immune activation processes. Accordingly, these findings do not fully distinguish transformation-specific biology from inflammation-associated responses. Instead, they indicate that epithelial–immune interactions differ between OED lesions with distinct clinical trajectories, potentially representing an early feature of disease evolution.

Consistent with this interpretation, pathway analyses suggest enrichment of immune-related processes, including interferon and cytokine signaling, while downregulated genes were associated with keratinization and epithelial differentiation. Given the limited DEG set, these results should be interpreted cautiously, as pathway enrichment may reflect overrepresentation of interferon-related genes rather than a defined regulatory program. Accordingly, pathway and network analyses are presented as exploratory findings.

The absence of significant immune DEGs in this study should not be interpreted as reduced or static immune involvement in the OED-T vs OED-NT comparison. Rather, it likely reflects a combination of limited detection sensitivity, cellular heterogeneity within CD45^+^ immune-enriched regions, and the targeted nature of the CTA panel. The segmentation strategy captures broad immune-associated compartments rather than specific immune cell subtypes, therefore limiting resolution.

Additional enrichment signals related to myeloid-associated and amyloid-related pathways were observed. Myeloid-derived suppressor cells (MDSCs) are known regulators of the tumor microenvironment and have been previously reported to be elevated in high-grade OED lesions (34). While our results are consistent with potential involvement of myeloid-associated signaling, they should be interpreted as indicative of broader immune-related activity rather than definitive evidence of MDSC involvement. Enrichment of amyloid-related pathways may also reflect underlying inflammatory processes. Myeloid cells contribute to systemic inflammatory responses, including the production of serum amyloid A (SAA), which has been associated with amyloid deposition in the upper aerodigestive tract (35). However, given the targeted nature of the gene panel and the limited number of DEG, the results should be interpreted cautiously.

Transcription factor analysis identified RELA (a subunit of the NF-κB complex) and RFX5 as potential upstream regulators. RELA is involved in inflammatory signaling and stress-response signaling and has been implicated in epithelial–mesenchymal transition, proliferation, and survival, while RFX5 regulates MHC class II expression and antigen presentation (36–40). Both factors are associated with genes identified in this study. f.

Comparisons across other groups revealed more extensive transcriptional differences, suggesting that molecular changes are more pronounced across larger biological transitions. In contrast, the subtle differences between OED-T and OED-NT highlight the challenge of identifying early transformation-associated signals within histologically similar lesions.

Several limitations should be acknowledged. The modest sample size limits statistical power, particularly for low-abundance transcripts. The use of a targeted ~1, 800-gene panel restricts transcriptomic coverage and may bias pathway interpretation. Downstream pathway and network results stemmed from exploratory analysis of a small DEG set and should not be interpreted as evidence of coordinated biological programs. Spatial segmentation provides only coarse compartment-level resolution, and each compartment likely contains heterogeneous cell populations. In addition, classification of OED-NT was based on a minimum two-year clinical follow-up period and may include lesions that have not yet progressed. Finally, exploratory analyses using nominal p-values introduce the potential for false positives.

Future studies incorporating larger cohorts, full-transcriptome approaches, and higher-resolution spatial or single-cell methods will be essential to refine these findings. In addition, orthogonal validation studies are currently underway using higher-resolution spatial transcriptomics (e.g., Xenium and Stereo-seq) and spatial high-plex proteomics (e.g., COMET) to further characterize these observations.

Overall, this study provides spatially resolved, exploratory insights into molecular differences associated with divergent clinical outcomes in OED, highlighting potential roles for immune-associated transcriptional activity in early disease stages.

## 5 Conclusion

In this study, we applied spatial transcriptomics to investigate molecular differences associated with malignant transformation in oral epithelial dysplasia. We identified a limited set of epithelial genes differentially expressed between OED-T and OED-NT, reflecting subtle transcriptional differences between clinically distinct but histologically similar lesions. The identified genes, including *B2M*, *STAT1*, and *CD74*, are associated with antigen presentation and immune signaling pathways.

Overall, this work highlights the potential of spatial transcriptomics to capture early, context-dependent molecular changes in OED. Further validation and higher-resolution studies will be required to determine the role of these signals in malignant progression and their potential utility as biomarkers for early detection.

## Data Availability

The datasets generated for the current study have been deposited in the NCBI Gene Expression Omnibus (GEO) public repository and are publicly available under accession number GSE324769. The dataset can be accessed at: https://www.ncbi.nlm.nih.gov/geo/query/acc.cgi?acc=GSE324769.
