# Supplementary material for: Spatial transcriptomics reveals molecular differences associated with malignant transformation in oral epithelial dysplasia
Source: Front Immunol. 2026 Jul 10;17:1817749. doi: 10.3389/fimmu.2026.1817749 (PMC13395910; doi:10.3389/fimmu.2026.1817749)

Supplementary Materials 1. Patient demographic and clinical information of the study cohorts.

M/F	BY	Biopsy Location	Histological Diagnosis	Clinical Description
**OED-T**				
F	1983	lingual #28-29	VHK with mild OED	leukoplakia
F	1934	left cheek, buccal and lingual upper left	VHK with moderate OED	multiple leukoplakia
M	1982	lateral tongue	Moderate to severe OED	not given
M	1957	gingiva papilla	Severe OED	erythroleukoplakia
M	1951	maxillary gingiva	VHK with severe OED	leukoplakia
M	1957	ventral tongue mucosa	Severe OED	multiple leukoplakia
M	1967	left tongue	Moderate OED	leukoplakia
**OED-NT**				
M	1939	#9 extraction site	Atypical EH w severe OED	mixed erythroleukoplakia
M	1952	lateral posterior 1/3 tongue	Moderate OED	hyperkeratotic lesion, leukoplakia
F	1945	retromolar pad area	HK & Acan w/moderate OED	Not given
F	1930	lateral/ventral tongue	Moderate to severe OED	leukoplakia
F	1945	buccal vestibule	VHK with moderate OED	not given
F	1963	ventral surface tongue	Mild to moderate OED	leukoplakia
F	1959	gingiva and mucosa	Atypical epithelium hyperplasia w ED	erythroleukoplakia
F	1945	buccal gingiva	Severe OED	raised roughened leukoplakia
**Benign**				
F	1974	buccal mucosa	HK & A	leukoplakia
M	1975	retromolar pad	HK & A	leukoplakia
F	1958	lateral border tongue	HK & A	leukoplakia
M	1971	anterior vestibule	HK & A	multiple leukoplakias
M	1992	floor of mouth	HK	leukoplakia
M	1992	retromolar pad	HK & A	leukoplakia
F	1955	lateral border tongue	HK & A	ulceration
M	1938	buccal gingiva	HK & A	small nodule on buccal gingiva
M	1956	lingual gingiva	HK & A	not given
F	1961	palate	HK & A	ulceration+ erythroleukplakia
M	1965	hard palate	HK & A	irregularity
M	1941	palatal gingiva	HK & A	leukoplakia
F	1948	lingual gingiva	HK & A with ulceration	ulcerated leukoplakia
**OSCC**				
M	1956	right cheek	Well diff. OSCC	ulcerated
F	1927	posterior vestibule	Moderately diff. OSCC	eroded

F	1941	attached gingiva	Well diff. OSCC	erythroplakia
M	1949	buccal attached gingiva	Moderately to well diff. OSCC	rough raised
M	1955	gingiva by tooth #13	Superficial Invasive OSCC	irregular rough lesion
F	1934	buccal mucosa	In situ OSCC	ulceration
F	1942	lateral tongue	Moderately to well diff. OSCC	ulceration
M	1935	buccal mucosa, mucosa of tuberosity	Moderately papillary SCC	rough spots w raised border
F	1931	attached gingiva	Moderately to well diff. SCC	erythroplakia
F	1961	lateral tongue	Well diff. OSCC	ulceration
M	1942	mandibular alveolar vestibule	Moderately to well diff. OSCC	ulceration
M	1931	hard palate	Moderately to poorly diff. OSCC	raised lesion
M	1955	interproximal papilla and slight palatal	Moderately diff. SCC	erythroplakia
F	1952	lateral tongue	Moderately to well diff. OSCC	firm, raised lesion

F: female; M: male; BY: birth year; OED: oral epithelial dysplasia; OSCC: oral squamous cell carcinoma; VHK: verrucous hyperkeratosis; HK: hyperkeratosis; A: acanthosis; diff.: differentiated.

Bioinformatic analysis of additional comparison group (Benign, OED, OSCC)

To provide biological context for the primary OED-T vs. OED-NT analysis presented in the main manuscript, we also performed additional comparisons across broader clinical stages of oral carcinogenesis, including OED vs. benign, OSCC vs. benign, and OSCC vs. OED. These analyses were intended to establish a reference framework for molecular changes associated with progression from benign oral tissue to dysplasia and carcinoma.

Compared with the OED-T vs. OED-NT comparison, substantially larger DEG sets were identified in these analyses (66, 107, and 10 DEGs for OED vs. benign, OSCC vs. benign, and OSCC vs. OED, respectively; **Supplementary Material 2**). Correspondingly, more connected PPI networks were observed in both epithelial and immune-enriched regions (**Supplementary Materials 3-4**). Compared to OED vs. benign and OSCC vs. benign comparisons, the OSCC vs. OED comparison generated relatively limited DEGs and less connected network structure.

Pathway enrichment analyses (**Supplementary Materials. 5-15**) were conducted using Benjamini–Hochberg (BH) correction (adjusted $p < 0.05$) to provide a more stringent reference analysis for these histopathologically distinct groups.

The OED vs. Benign comparison captured broader molecular changes potentially associated with the transition from healthy oral tissue to dysplasia. Enriched pathways were primarily related to immune activation, antigen processing and presentation, interferon signaling, and responses to infection. GO analysis also identified membrane-associated cellular components, including vesicles and lysosomal structures, suggesting alterations in immune-related cellular organization during early dysplastic change.

Similarly, the OSCC vs. Benign comparison revealed more extensive transcriptional differences potentially associated with malignant transformation. In epithelial regions, enriched pathways included immune modulation, cytokine signaling, and MHC-mediated antigen presentation. GO and pathway analyses also highlighted membrane-associated structures and protein complex binding functions, including pathways involving B2M and interferon-related signaling.

Importantly, several biological themes identified in these broader, FDR-corrected comparisons overlapped with findings from the exploratory OED-T vs. OED-NT analysis, including antigen presentation and interferon-related pathways. While this overlap does not validate transformation-specific mechanisms, it suggests that the exploratory findings are biologically consistent with broader molecular alterations observed during oral carcinogenesis.

Supplementary Materials 2. Differentially expressed genes (DEGs) across the three broader comparisons between Benign, OED, and OSCC.

**OED vs Benign**					**OSCC vs Benign**							**OSCC vs OED**			
Epithelial (37)			Immune (29)		Epithelial (68)				Immune (39)			Epithelial (8)		Immune (2)	
**UP**		**DOWN**	**UP**	**DOWN**	**UP**		**DOWN**		**UP**		**DOWN**	**UP**	**DOWN**	**UP**	**DOWN**
IFI27	LY6E	DUSP1	H3C10	S100A9	PARP9	STAT2	KRT10	IER3	TAP1	HLA-C	KRT14	TNFSF10	KRT1	SOD2	
STAT1	CXCL10	DSP	CXCL13	KRT5	IFI27	HLA-E	ANXA1	DSP	IFI6	CXCL9	KRT6A	LAMC2	PPL	HLA-C	
HLA-B	HLA-DRB3	PPL	TAP1	S100A8	IFI6	IFIT3	ARG1	PALMD	HLA-A	CCL18	COMP	ISG15	SPINK5		
B2M	ISG15	NFE2L2	STAT1	ANXA1	DTX3L	SLC2A1	RPS6	WEE1	HLA-B	LYZ	CXCL14	HLA-C	CALML3		
IFI6	IFIT3	SPINK5	MMP9	CALML5	BST2	LY6E	SERPINB5	CALML3	POU2AF1		A2M				
PARP9	CDH3	CD24	POU2AF1	KRT14	HLA-B	CD74	CALML5	PPL	STAT1		CD34				
HLA-A	CXCL9	RORA	CCL15	KRT6A	C1S	HLA-DRB	CRABP2	RORA	SOD2		ANXA1				
OAS3	GBP1	IER3	HLA-A	COMP	TYMP	HLA-DRA	FLG	KLF4	TYMP		CD9				
MX1		LAMB4	CXCL9	CD9	STAT1	MX1	RRAD	SPINK5	IRF4		KRT10				
TYMP			HLA-B	SFRP2	HLA-A	SOD2	JUP		IRF7		SPINK5				
CD74			HLA-F	DSP	PLAU	HLA-C	CD55		SLAMF7		DSP				
BST2			GNLY	KRT1	ISG15	LYZ	SERPINB2		HLA-F		ITGB4				
H3C10			CCL18	KRT10	B2M	GBP1	LAMB4		HSP90B1		KRT1				
HLA-DRA			TRBC1	CFD	IFITM1	SAMD9	BMP7		IRF8		KLF4				
HLA-F			B2M	KRT6B	OAS3	SERPING1	IL1RN		CXCL13		CFD				
HLA-E			TRBC2	KRT6C	TNFSF10	SAA1	NFE2L2		B2M		KRT6B				
TAP1					HLA-F	LAMC2	KRT1		GNLY		KRT6C				
HLA-DRB					APOL6	CXCL10	PKP3		MMP1						
IFITM1					CDH3	CXCL9	CD24		CD79A						
LYZ					TAP1		DUSP1		IGLL1						

UP: Upregulated, **DOWN:** Downregulated

Supporting Material 3. PPI networks generated by the STRING database using epithelial and immune-enriched DEGs from the three broader comparisons between Benign, OED, and OSCC.

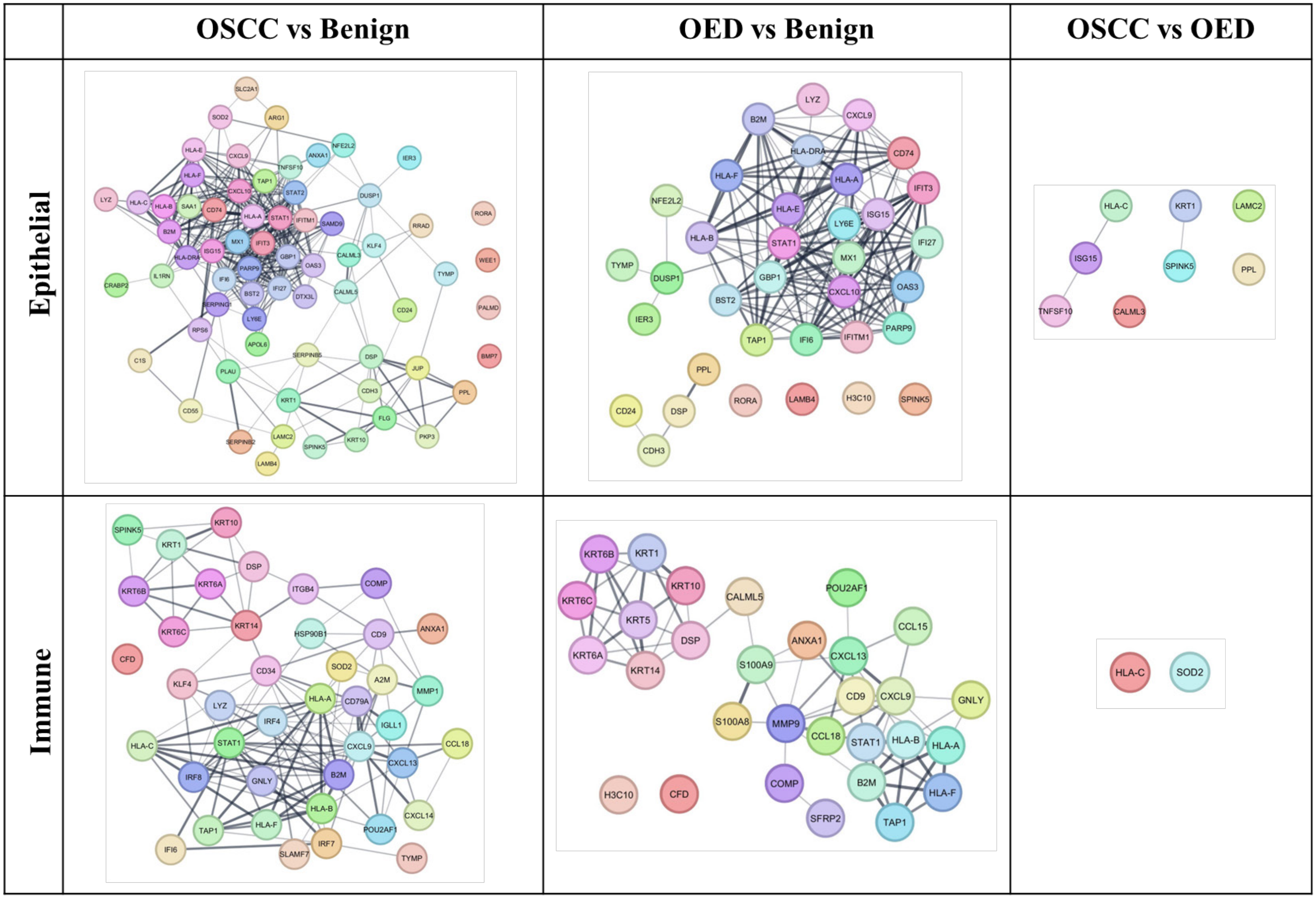

Supplementary Materials 4. Summary of number of nodes, edges, and MCODE scores for the three broader comparisons in epithelial and immune-enriched compartments.

Group	DEGs	Modules	MCODE		
			**Node**	**Cluster**	**Score**
**OSCC vs Benign (epithelial)**	68	5	14	83	12.769
			11	45	9
			5	10	5
			3	3	3
			3	3	3
**OED vs Benign (epithelial)**	37	2	13	77	12.833
			7	21	7
**OSCC vs Benign (immune)**	39	3	8	26	7.429
			5	7	3.5
			5	6	3
**OED vs Benign (immune)**	29	3	6	15	6
			6	11	4.4
			4	5	3.333

Supplementary Materials 5. GO analysis of epithelial DEGs upregulated in the OED vs. Benign comparison.

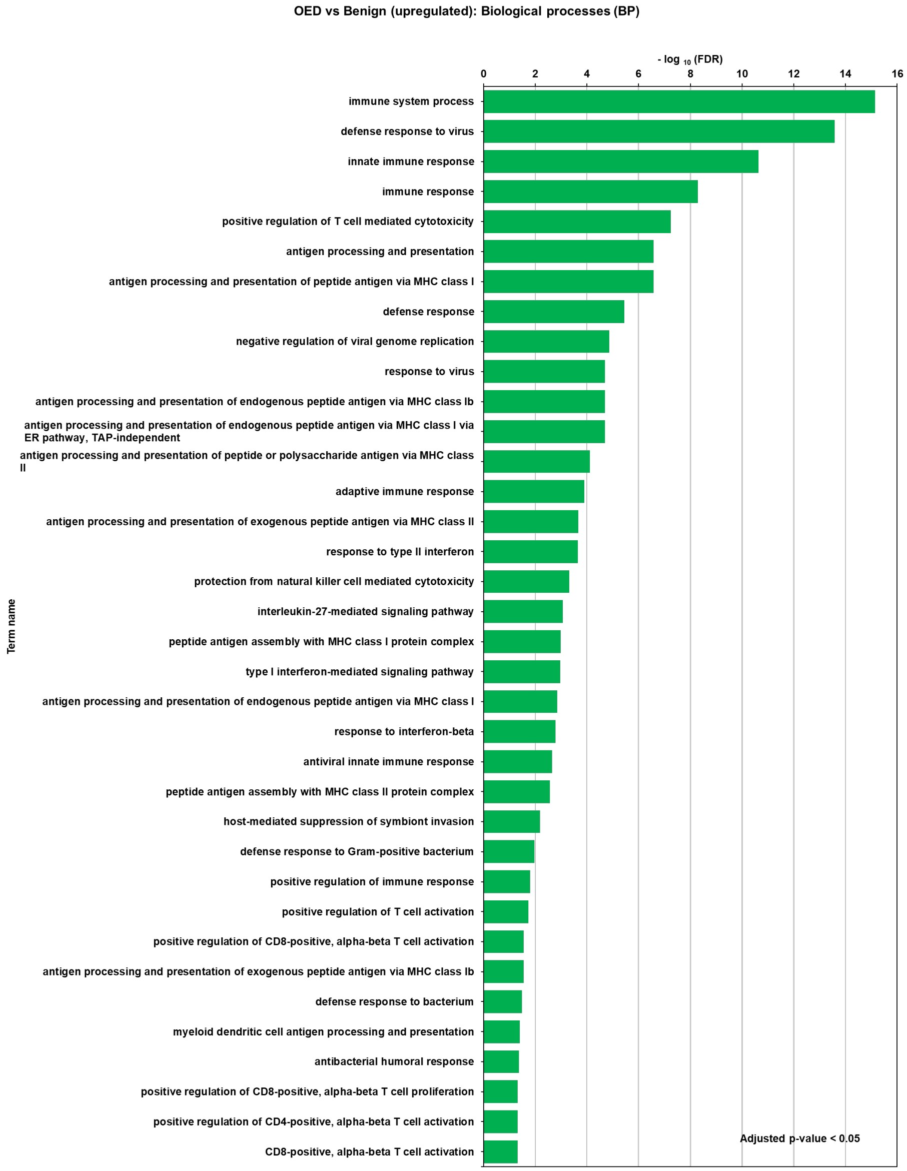

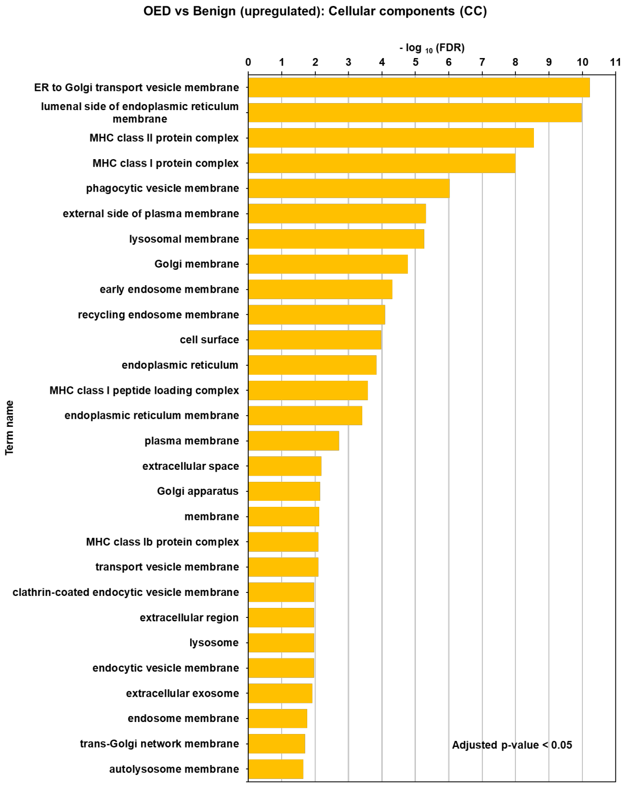

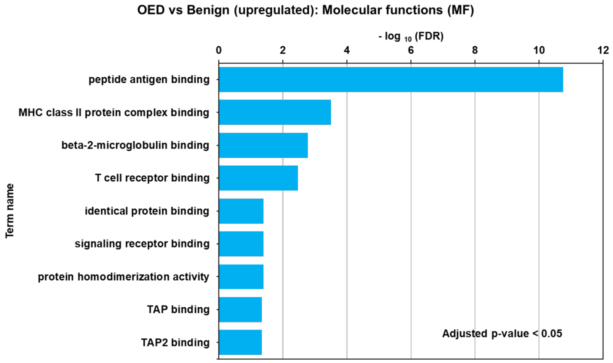

Supplementary Materials 6. Reactome and KEGG pathway enrichment analyses of upregulated epithelial genes in the OED vss Benign comparison.

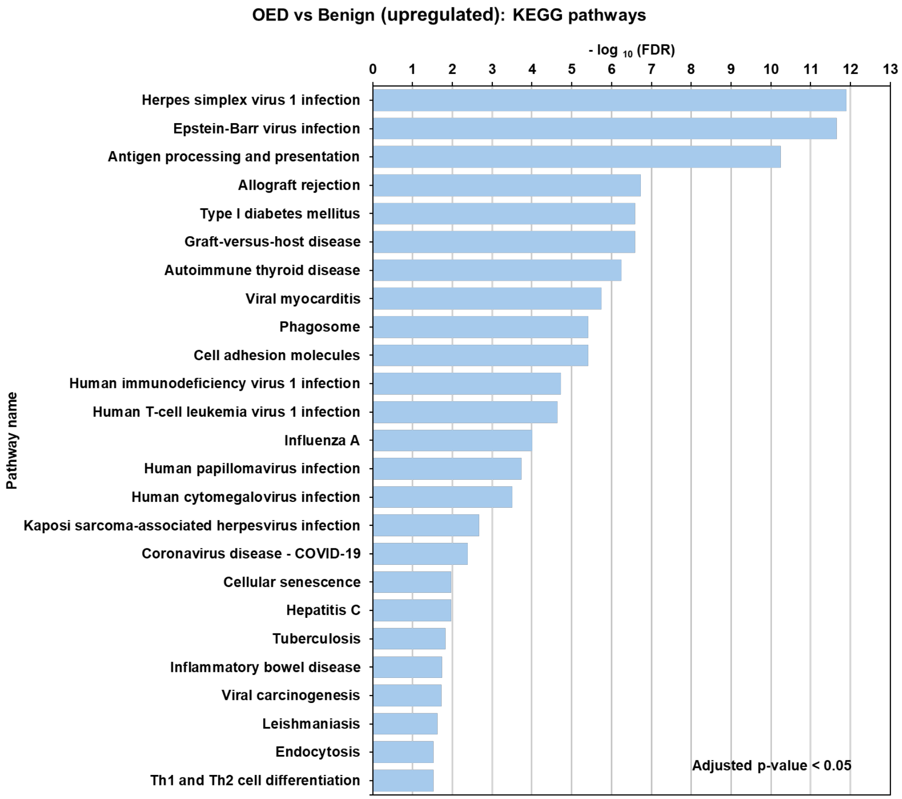

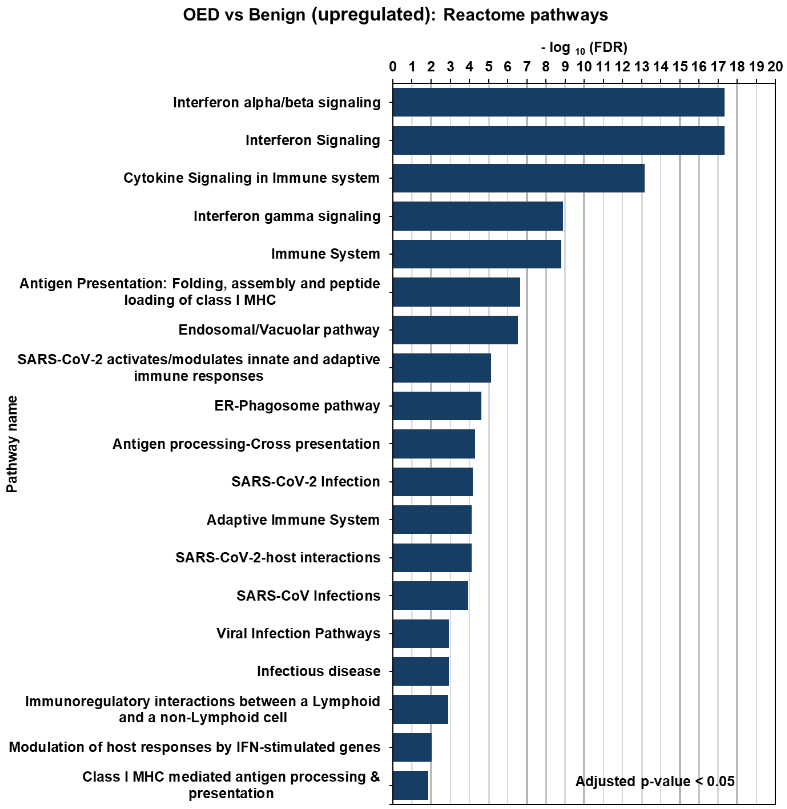

Supplementary Materials 7. GO analysis of epithelial DEGs upregulated in the OSCC vs. Benign comparison.

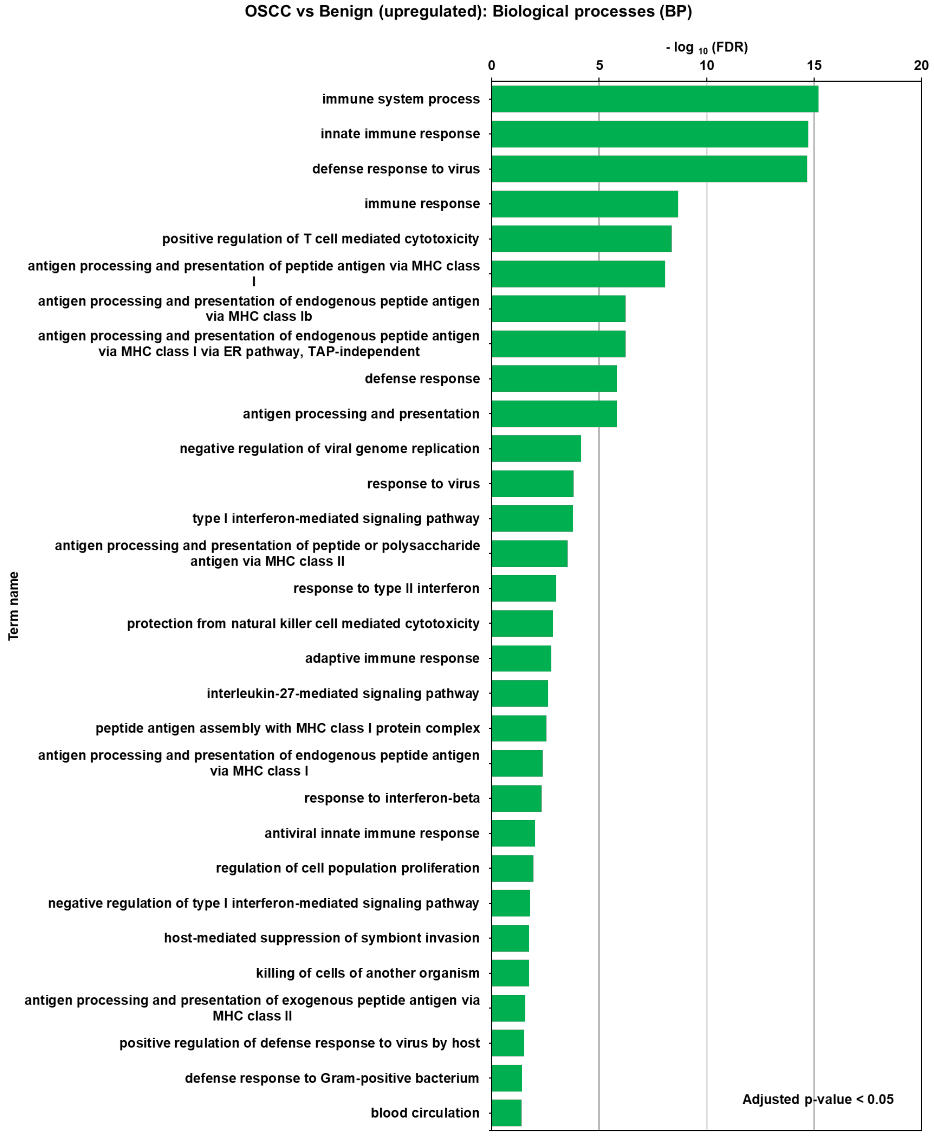

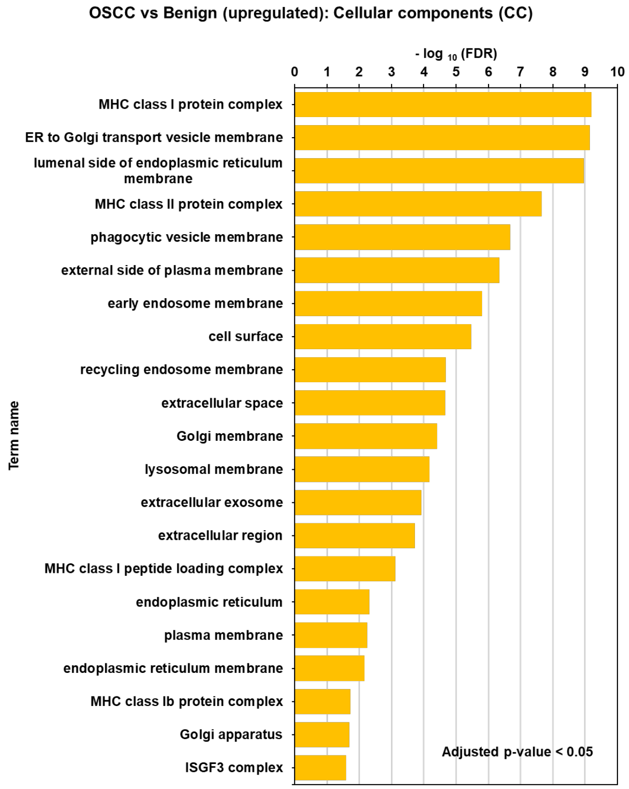

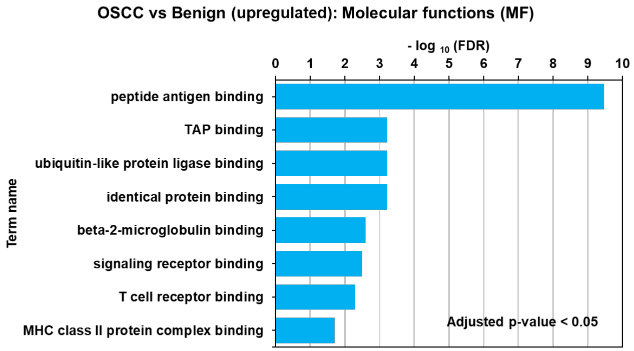

Supplementary Materials 8. Reactome and KEGG pathway enrichment analyses of upregulated epithelial genes in the OSCC vs. Benign comparison.

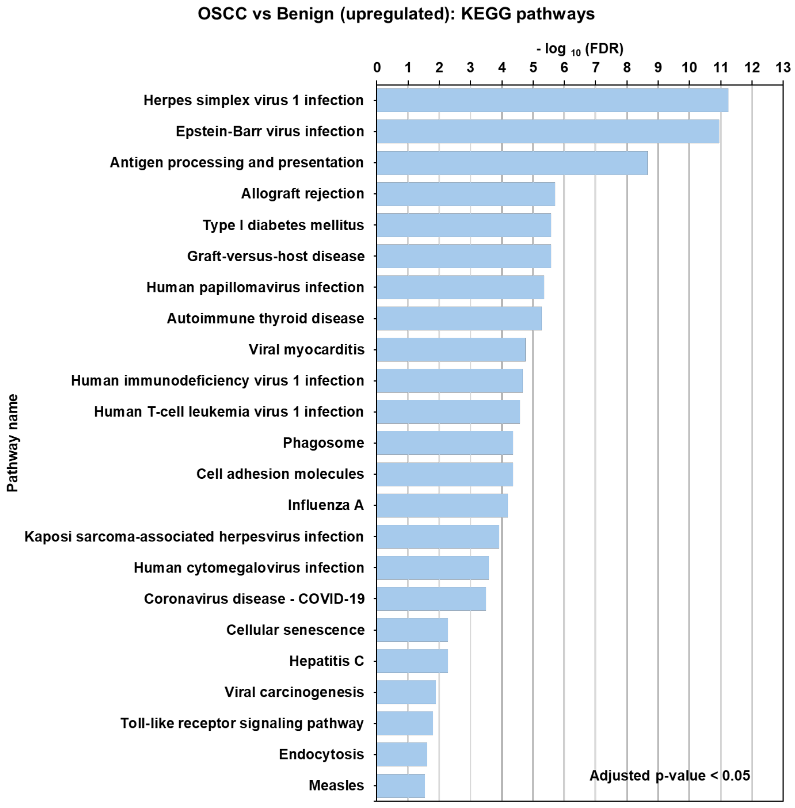

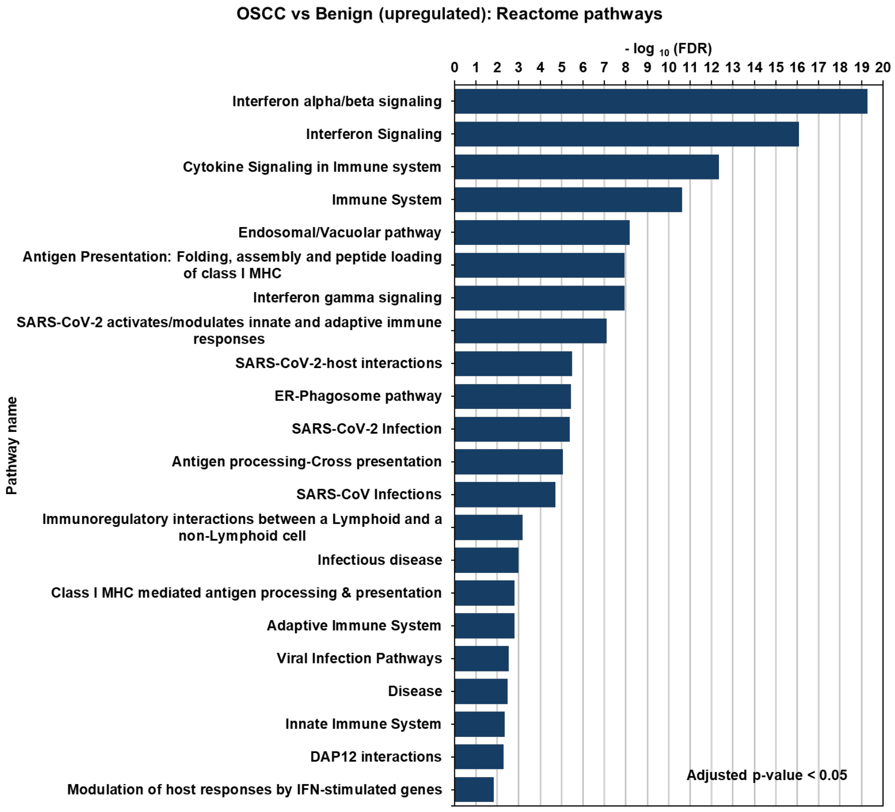

Supplementary Materials 9. GO, Reactome and KEGG pathway enrichment analyses of downregulated epithelial genes in the OSCC vs. Benign comparison.

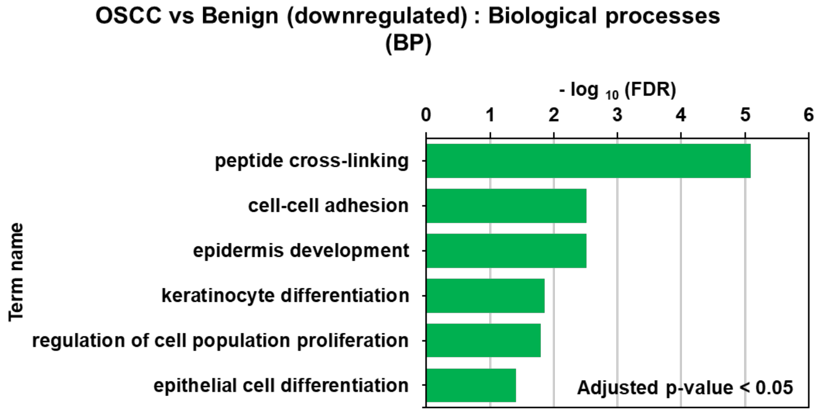

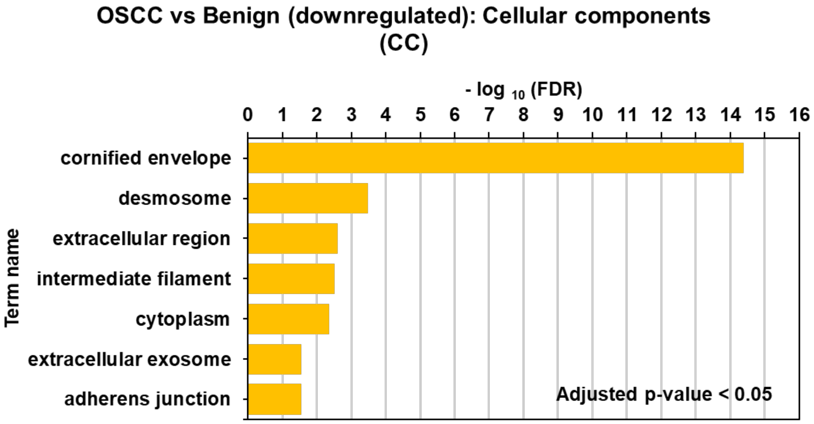

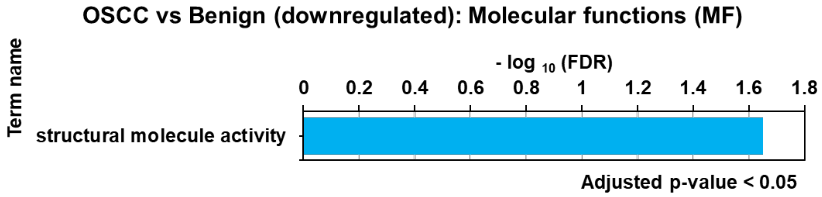

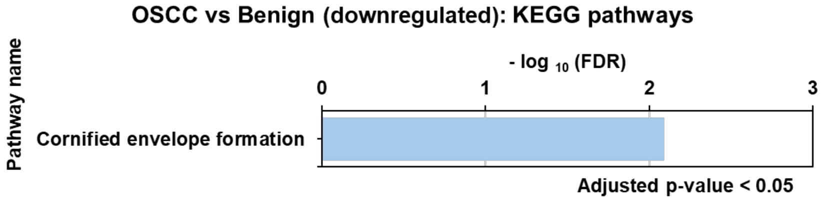

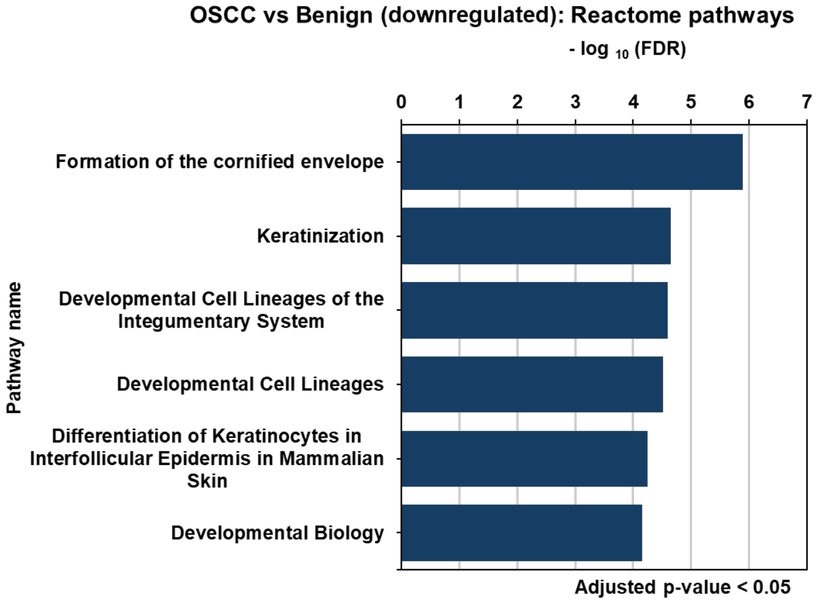

Supplementary Materials 10. GO analysis of upregulated immune-enriched DEGs in the OED vs. Benign comparison.

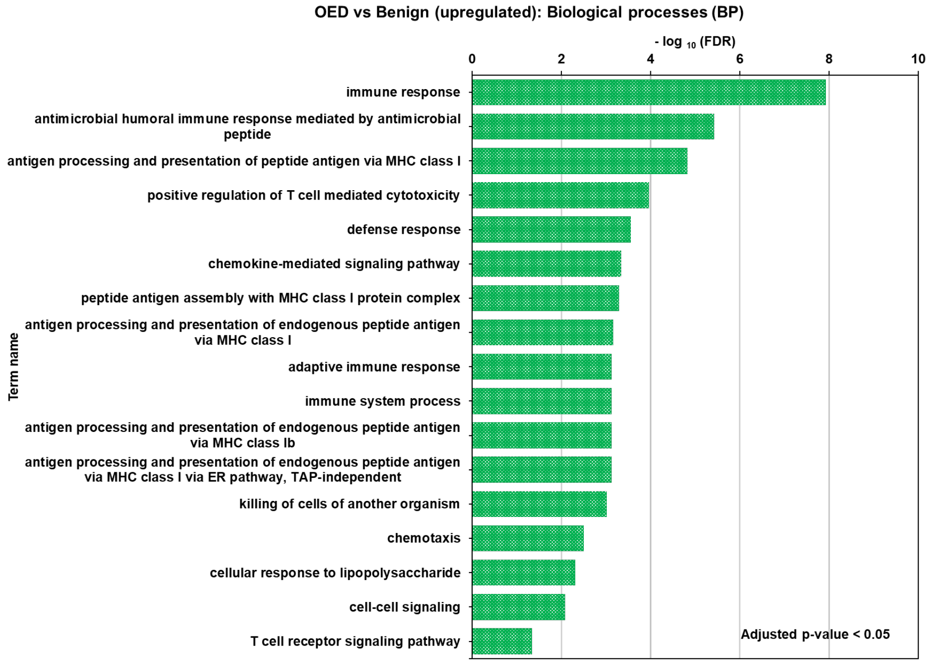

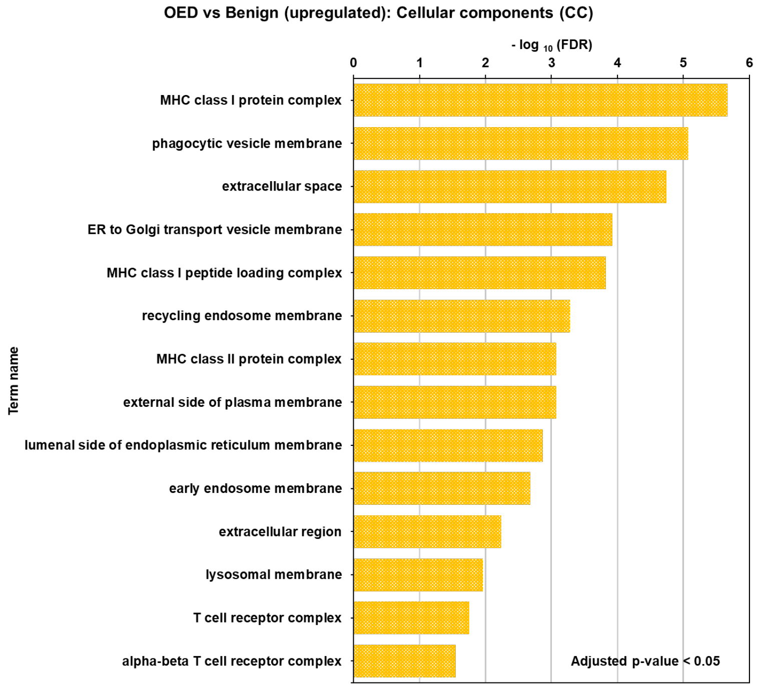

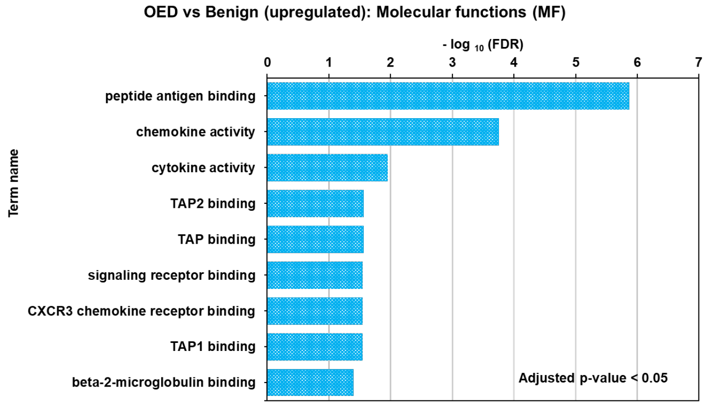

Supplementary Materials 11. Reactome and KEGG pathway enrichment analyses of upregulated immune enriched DEGs in the OED vs. Benign comparison.

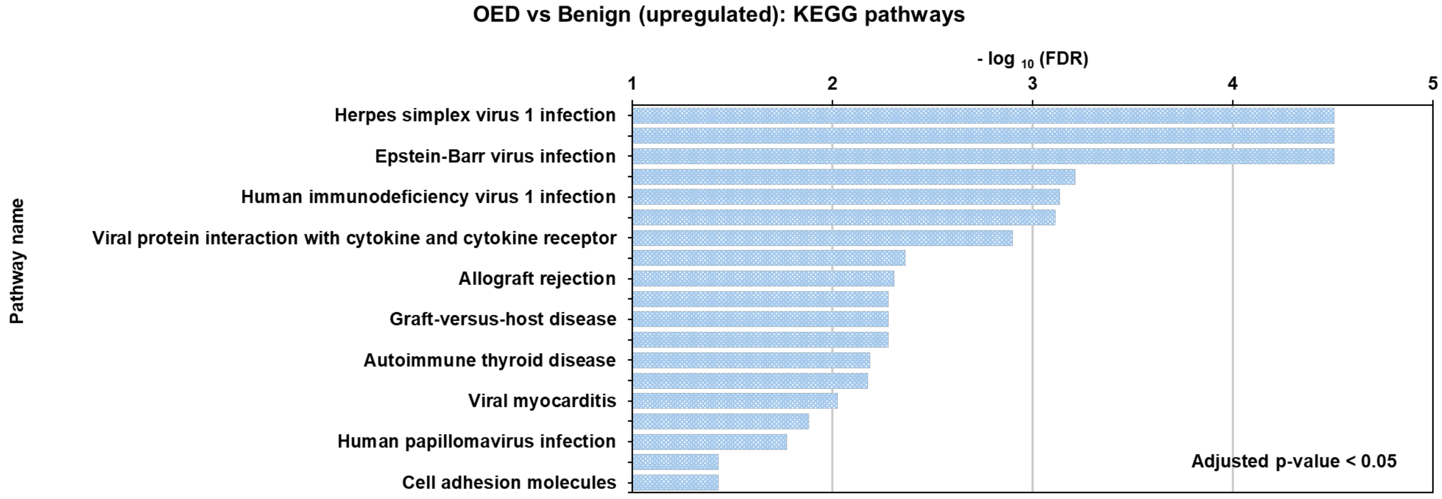

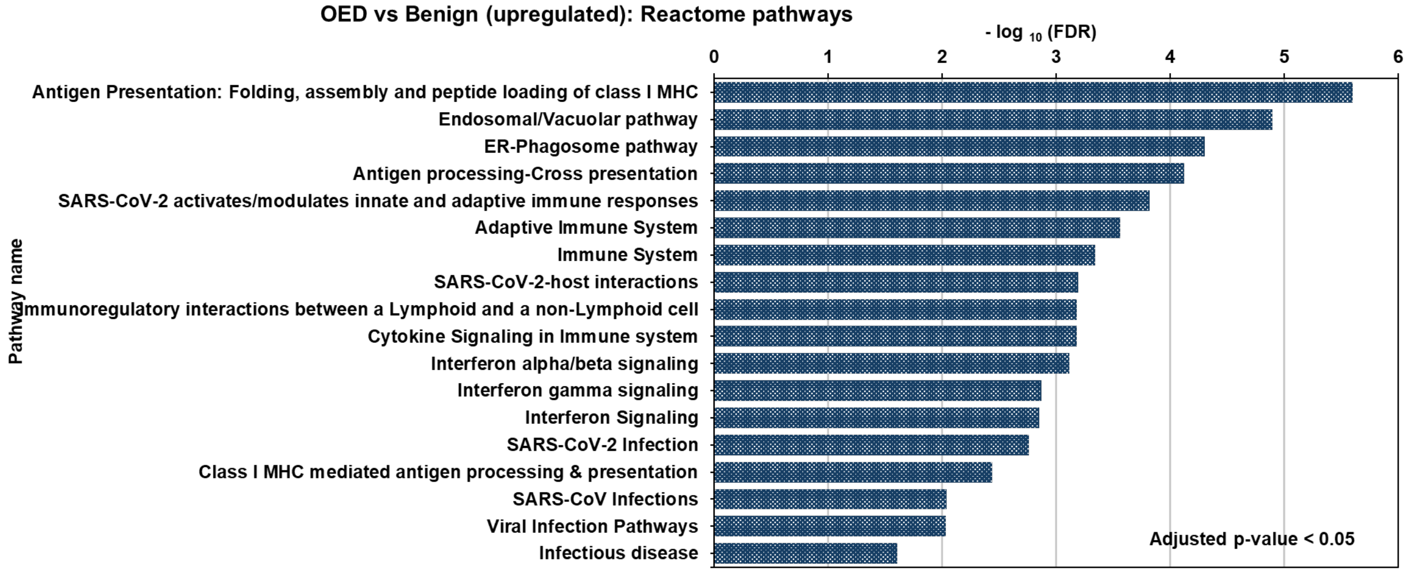

Supplementary Materials 12. GO, Reactome and KEGG pathway enrichment analyses of downregulated immune-enriched DEGs in the OED vs. Benign comparison.

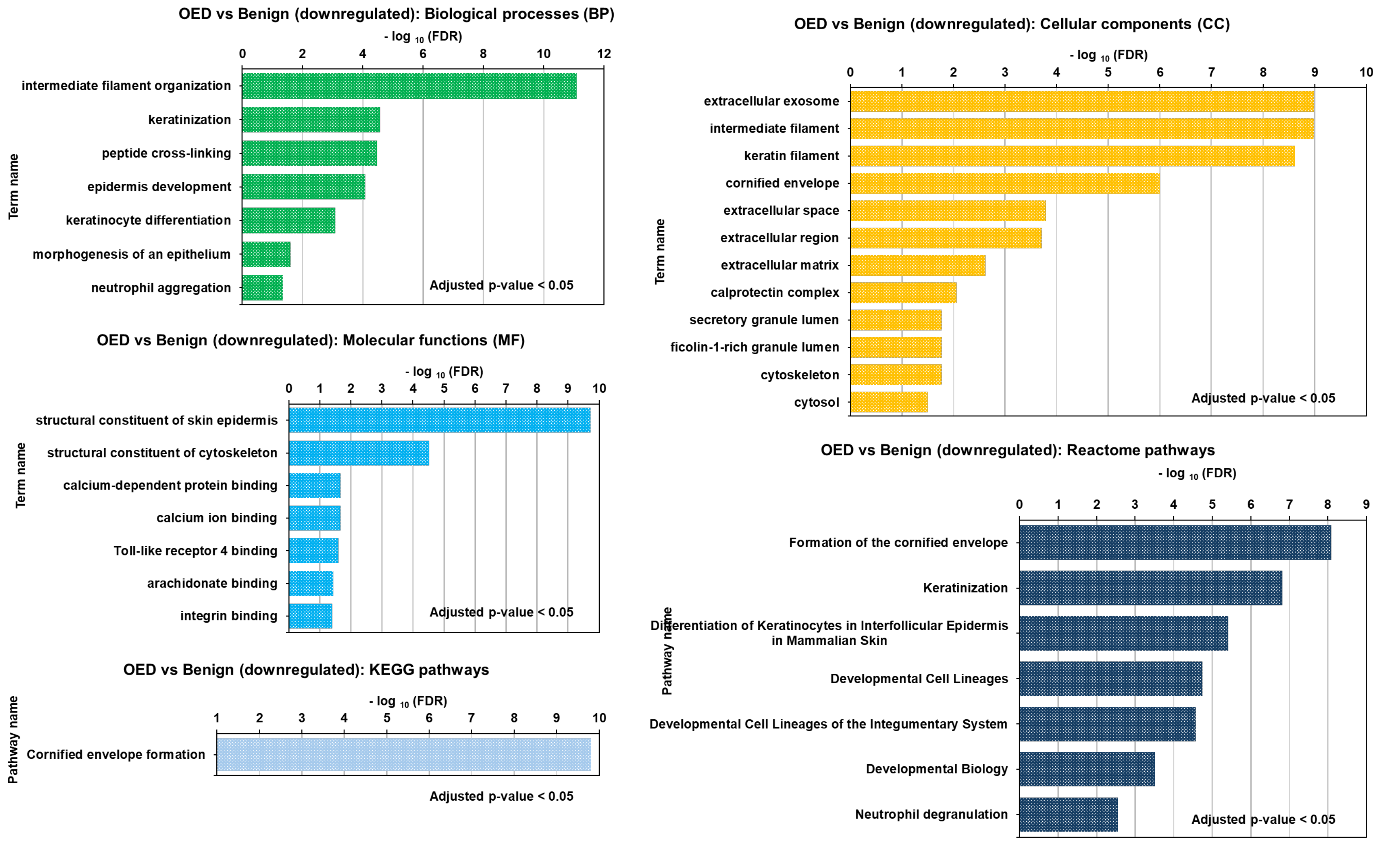

Supplementary Materials 13. GO analysis of upregulated immune-enriched DEGs in the OSCC vs. Benign comparison.

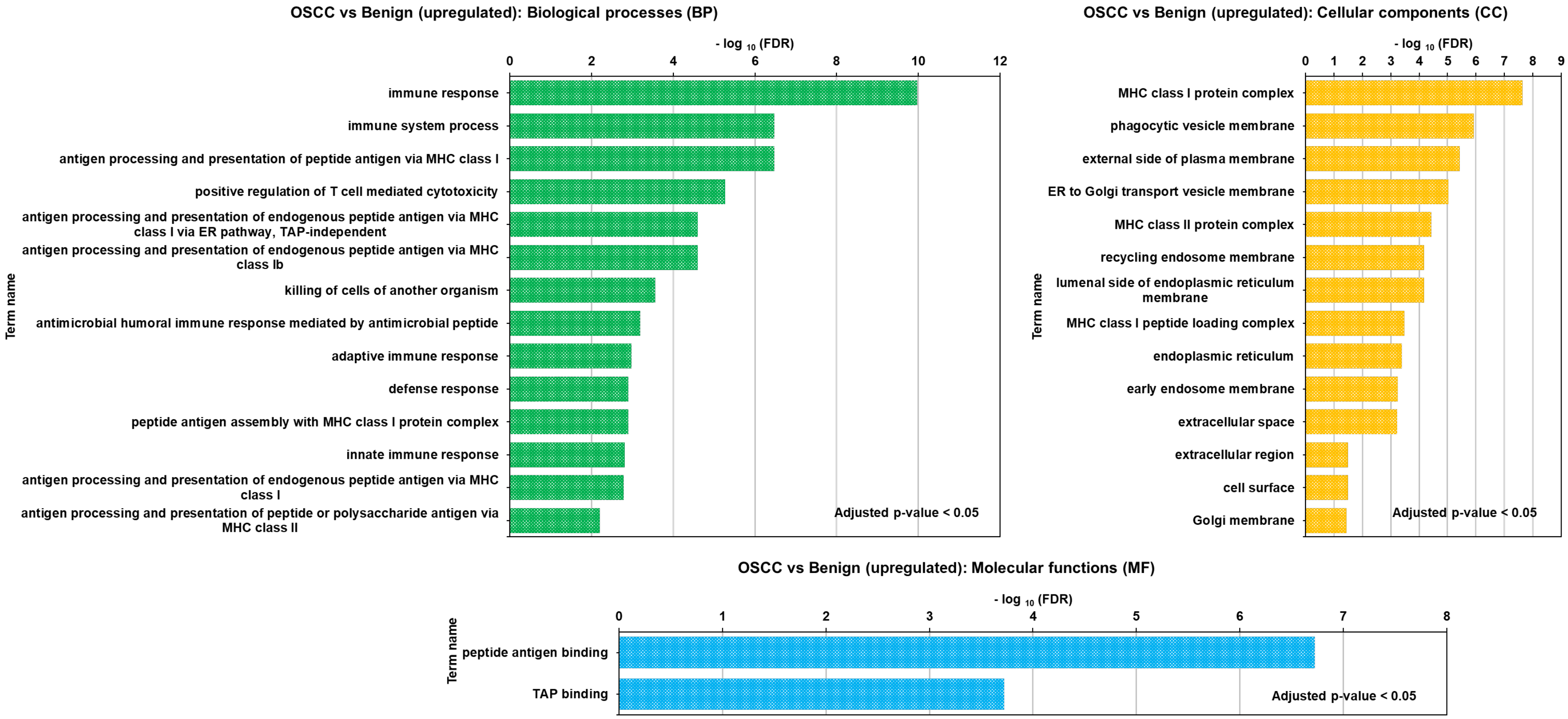

Supplementary Materials 14. Reactome and KEGG pathway enrichment analyses of upregulated immune-enriched DEGs in the OSCC vs. Benign comparison.

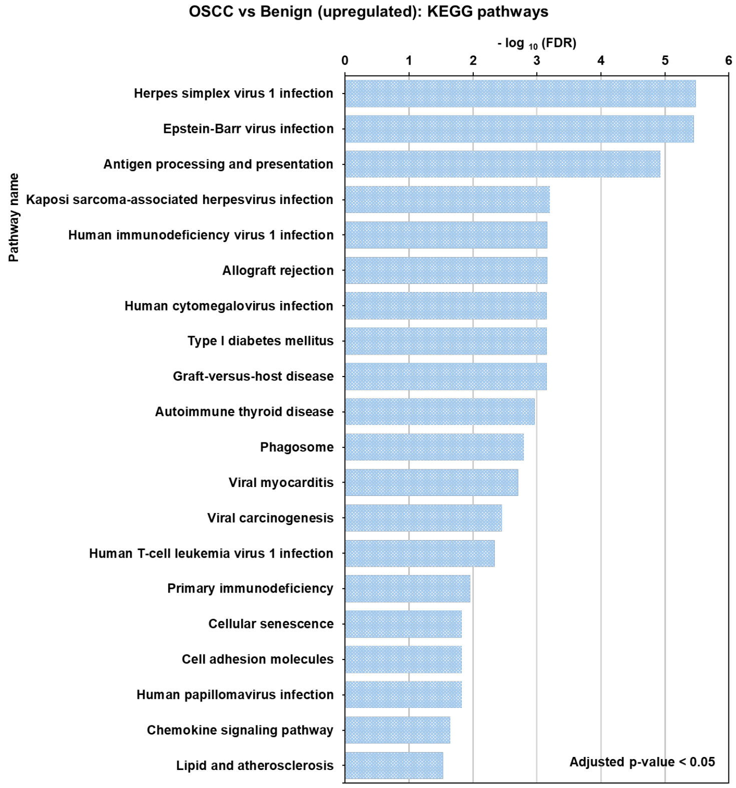

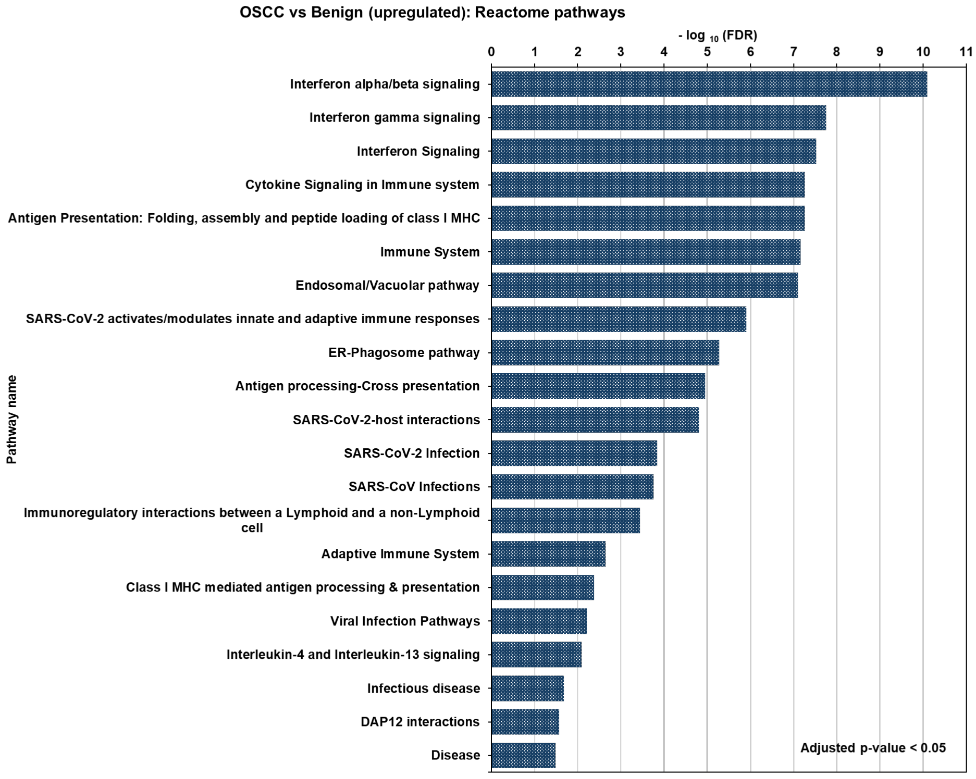

Supplementary Materials 15. GO, Reactome and KEGG pathway enrichment analyses of downregulated immune-enriched DEGs in the OSCC vs. Benign comparison.

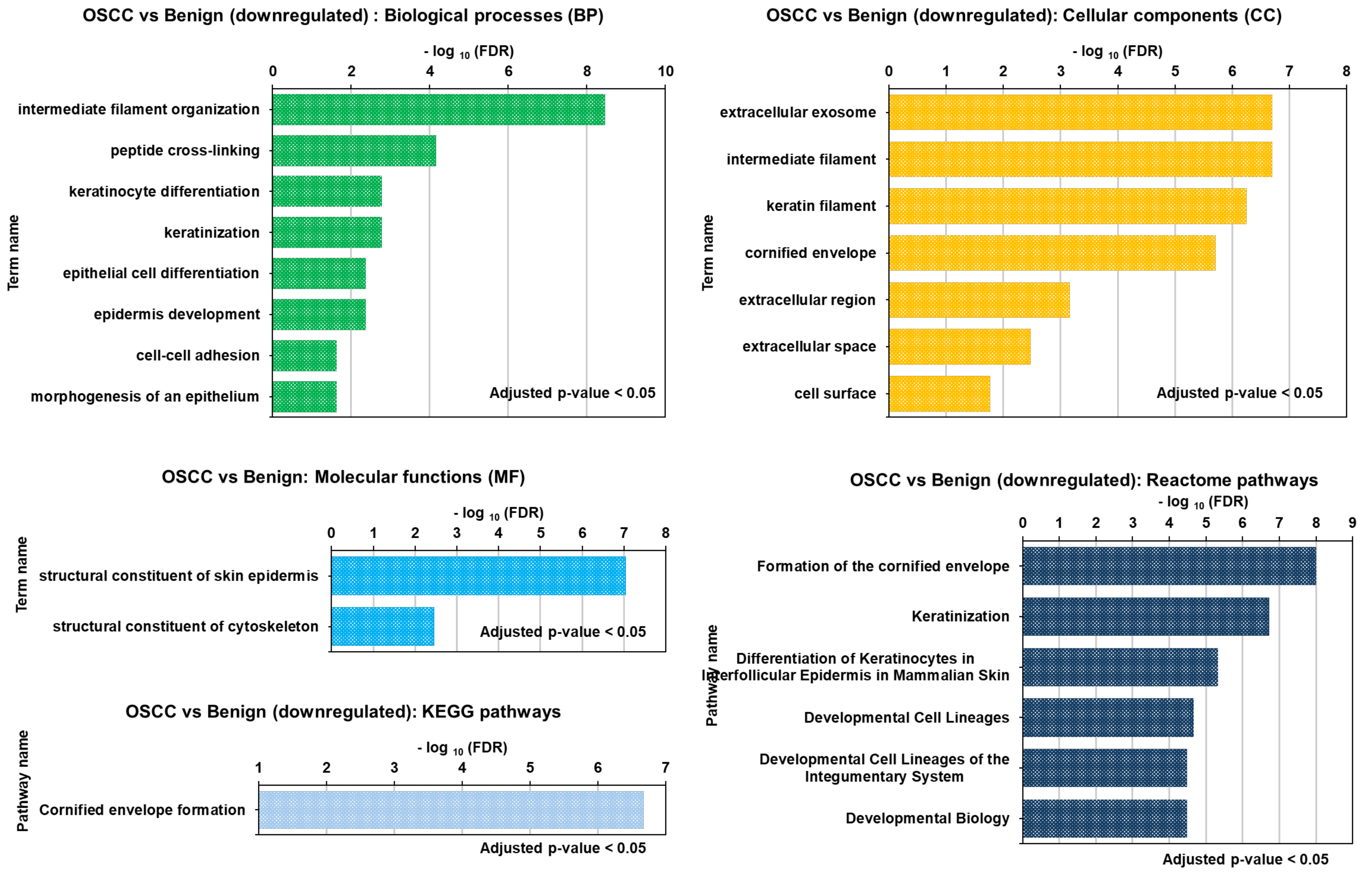

Supplement: Supplementary file 1 [file DataSheet1.pdf]
